# Supplementary material for: Furan-Conjugated Tripeptides as Potent Antitumor Drugs
Source: Biomolecules. 2020 Dec 16;10(12):1684. doi: 10.3390/biom10121684 (PMC7766758; doi:10.3390/biom10121684)
Supplement: Supplementary file 1 [file biomolecules-10-01684-s001.pdf]

Furan Conjugated Tripeptides as Potent Antitumor Drugs

Hunain Ali[1], Almas Jabeen[2], Rukesh Maharjan[1], Muhammad Nadeem-ul-Haque[1], Husena Aamra[1], Salma Nazir[1], Serab Khan[1], Hamza Olleik[3,4], Marc Maresca[3*] and Farzana Shaheen[1*]

[1]H. E. J. Research Institute of Chemistry, International Center for Chemical and Biological Sciences, University of Karachi, Karachi-75270, Pakistan.
[2]Dr. Panjwani Center for Molecular Medicine and Drug Research, International Center for Chemical and Biological Sciences, University of Karachi, Karachi-75270, Pakistan.
[3]Aix Marseille Univ, CNRS, Centrale Marseille, iSm2, 13397 Marseille, France.
[4]Department of Biology, American University of Beirut, Beirut-1107 2020, Lebanon.

Table of Contents

List of Figures		
**Figure No.**	**Content**	**Page No.**
S1	Structures of peptide conjugates 1-20	3
S2	UPLC Profile of Peptide Conjugates 1-10 in ACN:H_2O:TFA::60:40:0.08 at 215 nm	4-5
S3	UPLC Profile of Peptide Conjugates 11-18 and 20	6-8
S4	FABMS (Low Resolution Positive Mode) of Conjugate 1	11
S5	FABMS (High Resolution Positive Mode) of Conjugate 1	11
S6	^{1}H-NMR of Conjugate 1	12
S7	ESIMS (Low Resolution Positive Mode) of Conjugate 2	13
S8	FABMS (High Resolution Positive Mode) of Conjugate 2	13
S9	^{1}H-NMR of Conjugate 2	14
S10	ESIMS (Low Resolution Positive Mode) of Conjugate 3	15
S11	FABMS (High Resolution Positive Mode) of Conjugate 3	15
S12	^{1}H-NMR of Conjugate 3	16
S13	FABMS (Low Resolution Positive Mode) of Conjugate 4	17
S14	FABMS (High Resolution Positive Mode) of Conjugate 4	17
S15	^{1}H-NMR of Conjugate 4	18
S16	FABMS (Low Resolution Positive Mode) of Conjugate 5	19
S17	FABMS (High Resolution Positive Mode) of Conjugate 5	19
S18	^{1}H-NMR of Conjugate 5	20
S19	FABMS (Low Resolution Positive Mode) of Conjugate 6	21
S20	FABMS (High Resolution Positive Mode) of Conjugate 6	21
S21	^{1}H-NMR of Conjugate 6	22
S22	FABMS (Low Resolution Positive Mode) of Conjugate 7	23
S23	FABMS (High Resolution Positive Mode) of Conjugate 7	23
S24	^{1}H-NMR of Conjugate 7	24
S25	ESIMS (Low Resolution Positive Mode) of Conjugate 8	25
S26	FABMS (High Resolution Positive Mode) of Conjugate 8	25
S27	^{1}H-NMR of Conjugate 8	26
S28	FABMS (Low Resolution Positive Mode) of Conjugate 9	27
S29	FABMS (High Resolution Positive Mode) of Conjugate 9	27
S30	^{1}H-NMR of Conjugate 9	28
S31	FABMS (Low Resolution Positive Mode) of Conjugate 10	29
S32	FABMS (High Resolution Positive Mode) of Conjugate 10	29
S33	^{1}H-NMR of Conjugate 10	30
S34	ESI Spectra (Low Resolution) of Conjugate 4a	31
S35	ESI Spectra (Low Resolution) of Conjugate 4b	32
S36	Structural relationship between peptide conjugates 11-20	35
S37	ESI Mass spectrum for peptide conjugates 11-17, 19 (FAB-MS) and 20	36-39
List of Tables		
S1	^{1}H-NMR (600 MHz) and ^{13}C-NMR (150 MHz) data of conjugate 1-5 in DMSO-d_6	9
S2	^{1}H-NMR (600 MHz) and ^{13}C-NMR (150 MHz) data of conjugate 6-10 in DMSO-d_6	10
S3	TFA Cocktails for cleavage of peptide conjugates 1-20	33
S4	HPLC conditions for peptide conjugates 11-20	33
S5	Sequence and ESIMS (Low Resolution) and FABMS (High Resolution) of peptide conjugates 11-20	34

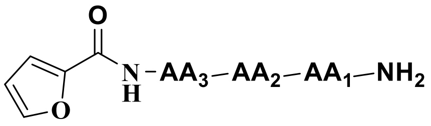

Conjugate No.	AA_3	AA_2	AA_1	Conjugate No.	AA_3	AA_2	AA_1
**1**	D-2-Nal	D-Phe	D-Ala	**11**	L-Tyr	D-Phe	D-Ala
**2**	L-2-Nal	L-Phe	L-Ala	**12**	D-Ala	D-Phe	D-Ala
**3**	L-2-Nal	L-Ala	L-Ala	**13**	D-Ala	D-Phe	Gly
**4**	L-2-Nal	L-Ala	L-Phe	**14**	D-Ala	D-Phe	L-Lys
**5**	L-2-Nal	L-2-Nal	L-Ala	**15**	D-Ala	L-2-Nal	D-Ala
**6**	L-Phe	L-2-Nal	L-Ala	**16**	D-Ala	L-Ile	D-Ala
**7**	L-Ala	L-2-Nal	L-Phe	**17**	D-Ala	L-Arg	D-Ala
**8**	L-Ala	L-Phe	L-2-Nal	**18**	D-Ala	D-Phe	-
**9**	L-2-Nal	L-Tyr	L-Ala	**19**	L-2-Nal	D-Phe	L-*N*-Me-Ala
**10**	L-2-Nal	L-Phg	L-Ala	**20**	L-2-Nal	D-Phe	L-Ala

Figure S1: Structures of peptide conjugates 1-20

Peptide Conjugate	UPLC Profile of Peptide Conjugates in ACN:H_2O:TFA::60:40:0.08 at 215 nm
1	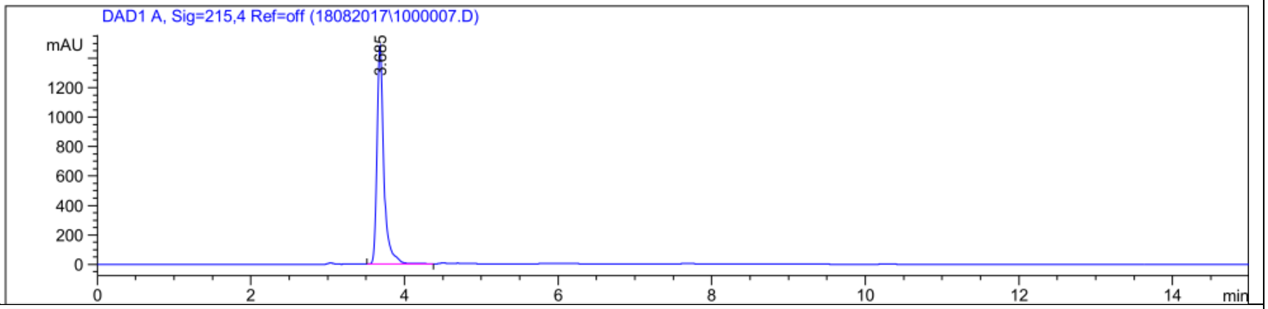
2	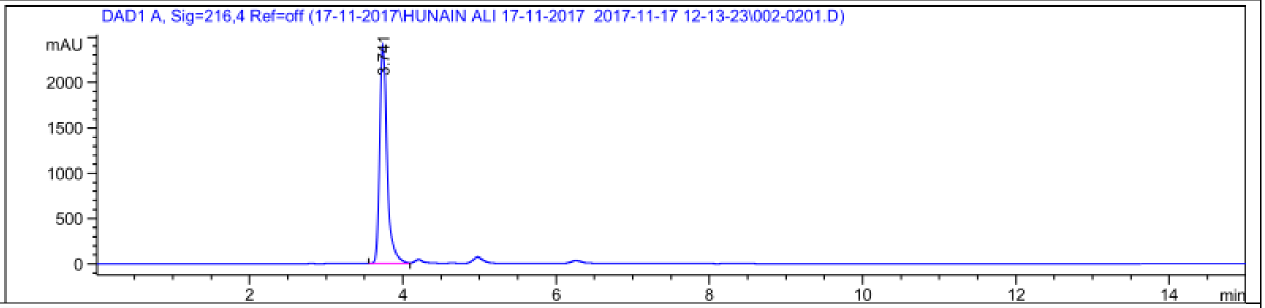
3	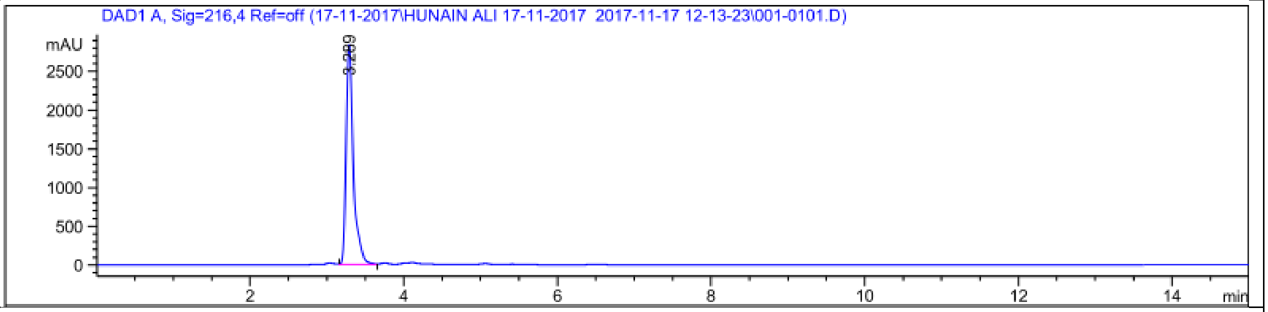
4	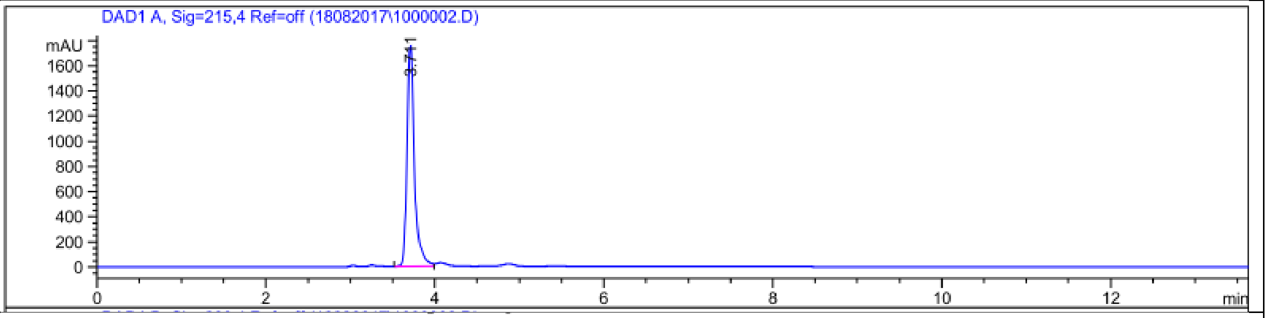
5	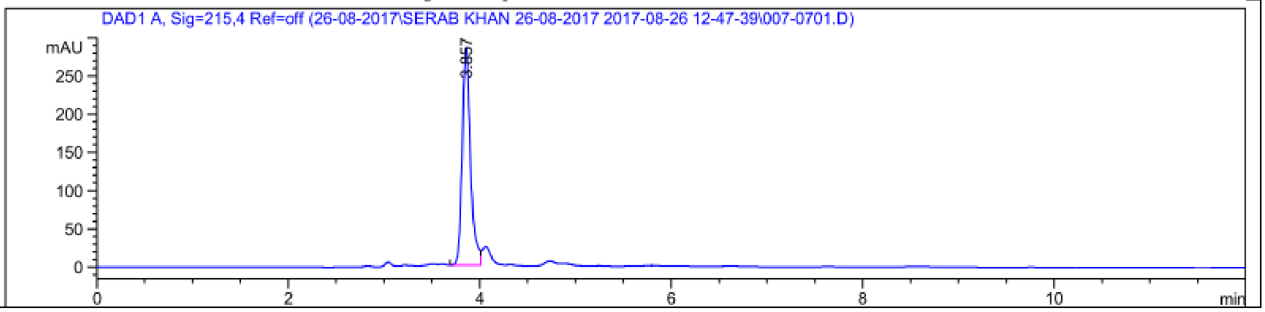

**6**	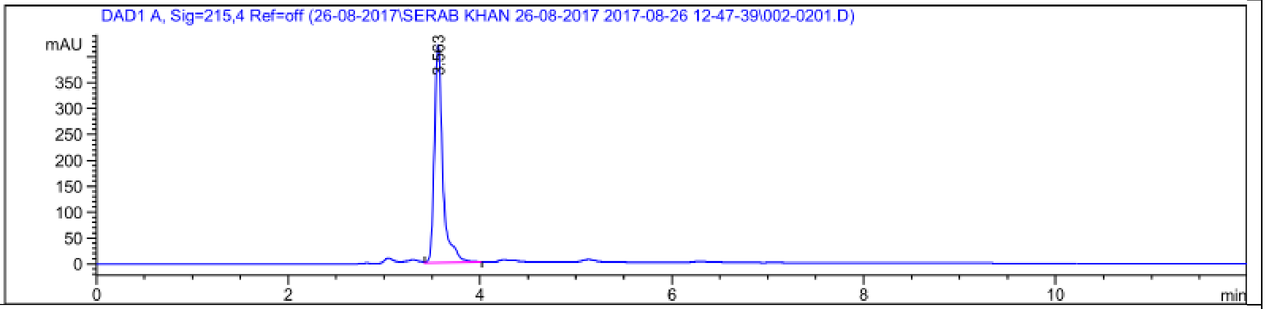
**7**	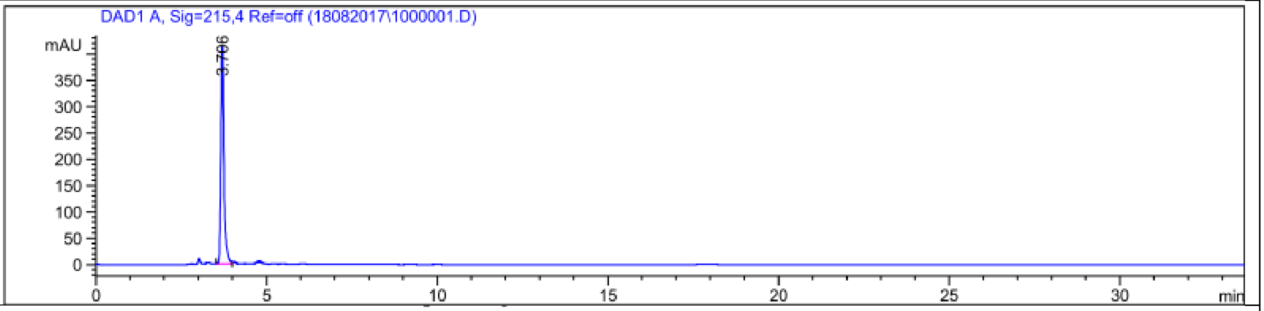
**8**	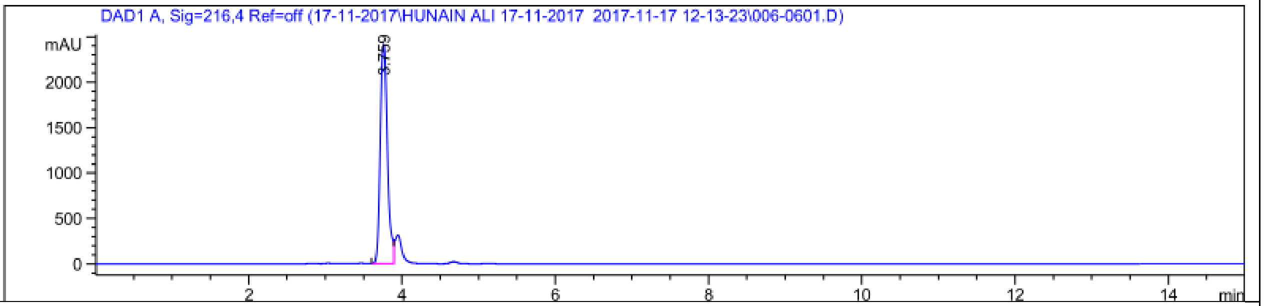
**9**	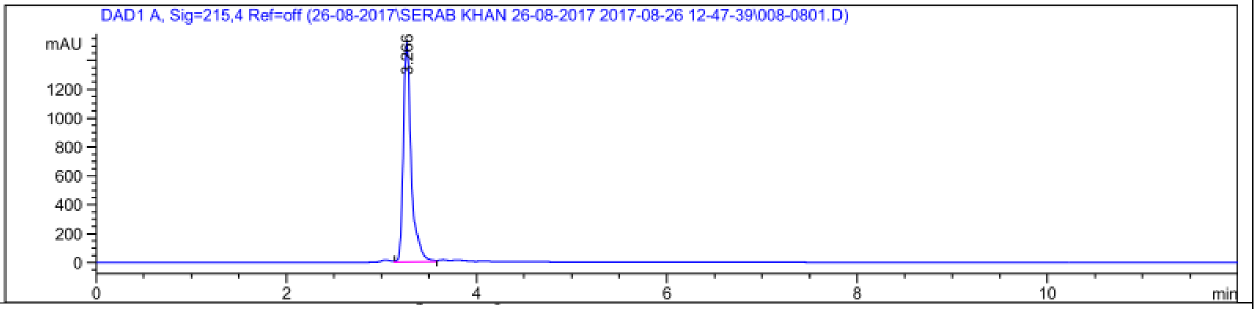
**10**	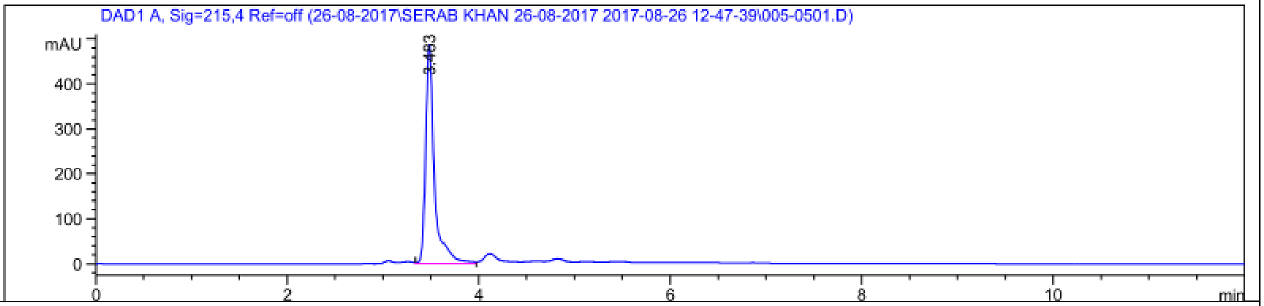

Figure S2: UPLC Profile of Peptide Conjugates 1-10 in ACN:H_2O:TFA::60:40:0.08 at 215 nm

Peptide Conjugate	UPLC Profile of Peptide Conjugates	Wavelength (nm)
**11**	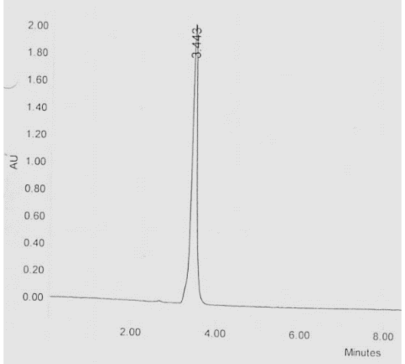	262.9
**12**	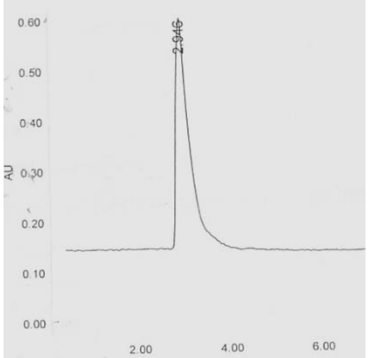	297.6
**13**	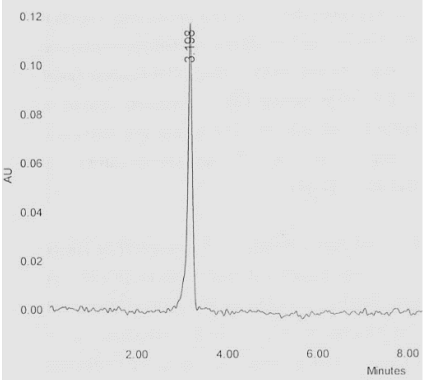	253.8
**14**	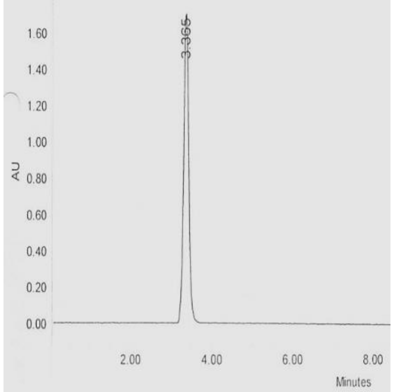	287.7

**15**	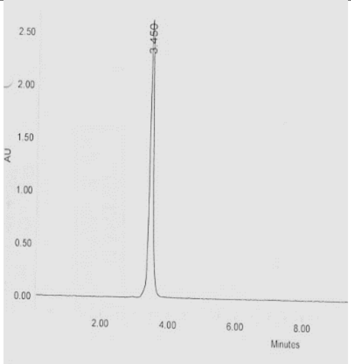	276.7
**16**	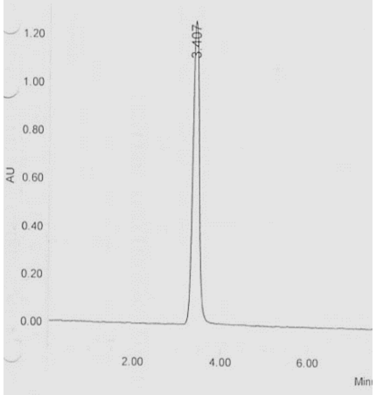	267.8
**17**	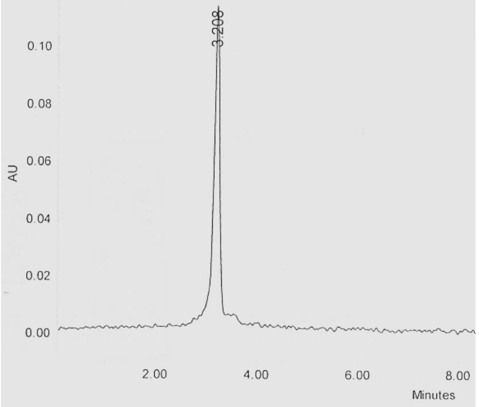	309.4
**18**	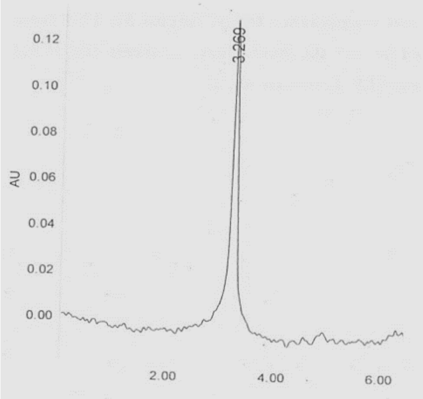	278.9

**20**	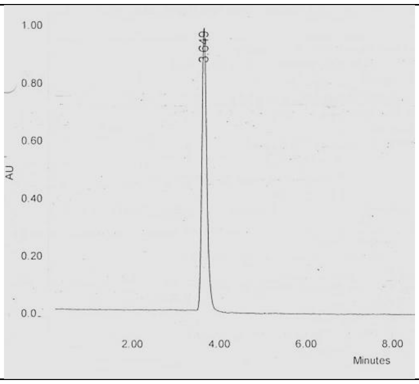	236.5
**Figure S3: UPLC Profile of Peptide Conjugates 11-18 and 20**		

Table S1. ^{1}H-NMR (600 MHz) and ^{13}C-NMR (150 MHz) data of conjugate 1-5 in DMSO-d_6

1

Residue	δ_H	δ_C
**D-Ala[1]**		
α	4.16 p (7.2 Hz)	48.3
CH_3	1.20 d (7.2 Hz)	18.0
NH	8.04 d (7.2 Hz)	-
C=O	-	174.3
NH_2	7.71 br. s	-
**D-Phe[2]**		
α	4.53 td (4.2, 8.4 Hz)	54.1
β, β′	3.1 dd (3, 13.2 Hz), 2.80 dd (9, 13.8 Hz)	37.5
1	-	137.7*
2, 6	7.15 m	126.5
3, 5	7.60 m	127.6
4	7.22 m	127.4
NH	8.24 d (7.8 Hz)	-
C=O	-	171.9
**D-2–Nal[3]**		
α	4.70 td (4.2, 9.6 Hz)	54.1
β, β′	3.10 dd (10.2, 13.2 Hz), 3.20 dd (4.2, 13.8 Hz)	37.3
1	7.38 m	125.5
2	-	137.7*
3	7.40 m	125.6
4	7.70 m	127.5
5, 8	7.19 m	128.1
6, 7	7.25 m	129.2
9	-	133.0
10	-	131.0
NH	8.27 d (8.4 Hz)	-
C=O	-	170.0
**2-Furoyl[4]**		
C	-	147.3
α	7.05 d (3.6 Hz)	114.6
β	6.53 dd (1.8, 3.6 Hz)	112.1
γ	7.76 br. s	145.3
C=O	-	157.7

2

Residue	δ_H	δ_C
**L-Ala[1]**		
α	4.16 p (7.2 Hz)	48.3
CH_3	1.20 d (7.2 Hz)	18.0
NH	8.04 d (7.2 Hz)	-
C=O	-	174.3
NH_2	7.71 br. s	-
**L-Phe[2]**		
α	4.53 td (4.2, 8.4 Hz)	54.1
β, β′	3.10 dd (3, 13.2 Hz), 2.80 dd (9, 13.8 Hz)	37.5
1	-	137.7*
2, 6	7.15 m	126.5
3, 5	7.60 m	127.6
4	7.22 m	127.4
NH	8.24 d (7.8 Hz)	-
C=O	-	171.9
**L-2–Nal[3]**		
α	4.70 td (4.2, 9.6 Hz)	54.1
β, β′	3.10 dd (10.2, 13.2 Hz), 3.20 dd (4.2, 13.8 Hz)	37.3
1	7.38 m	125.5
2	-	137.7*
3	7.40 m	125.6
4	7.70 m	127.5
5, 8	7.19 m	128.1
6, 7	7.25 m	129.2
9	-	133.0
10	-	131.0
NH	8.27 d (8.4 Hz)	-
C=O	-	170.0
**2-Furoyl[4]**		
C	-	147.3
α	7.05 d (3.6 Hz)	114.6
β	6.53 dd (1.8, 3.6 Hz)	112.1
γ	7.76 br. s	145.3
C=O	-	157.7

3

Residue	δ_H	δ_C
**L-Ala[1]**		
α	4.18 p (7.2 Hz)	48.9
CH_3	1.20 d (6.6 Hz)	18.2
NH	7.84 d (7.8 Hz)	-
C=O	-	175.6
NH_2	7.80 br. s	-
**L-Ala[2]**		
α	4.30 p (7.2 Hz)	49.3
CH_3	1.26 d (7.2 Hz)	18.5
NH	8.36 d (7.2 Hz)	-
C=O	-	172.8
**L-2–Nal[3]**		
α	4.71 td (4.2, 9 Hz)	54.7
β, β′	3.13 m, 3.18 m	37.8
1	7.38 m	125.5*
2	-	135.9
3	7.40 m	125.6*
4	7.70 m	127.5
5, 8	7.19 m	128.1
6, 7	7.25 m	129.2
9	-	133.0
10	-	131.0
NH	8.38 d (8.4 Hz)	-
C=O	-	170.0
**2-Furoyl[4]**		
C	-	147.3
α	7.05 d (3.6 Hz)	115.6
β	6.53 dd (1.8, 3.6 Hz)	112.7
γ	7.76 br. s	146.3
C=O	-	157.7

4

Residue	δ_H	δ_C
**L-Phe[1]**		
α	4.40 td (4.8, 8.4 Hz)	53.7
β, β′	3.00 dd (4.8, 13.8 Hz). 2.85 dd (8.4, 13.8 Hz)	37.5
1	-	137.7*
2, 6	7.80 m	127.5
3, 5	7.45 m	126.1
4	7.74 m	127.8
NH	7.83 m	-
C=O	-	172.8
NH_2	7.35 br. s	-
**L-Ala[2]**		
α	4.25 p (7.2 Hz)	48.7
CH_3	1.20 d 7.2 Hz)	18.0
NH	8.30 d (7.2 Hz)	-
C=O	-	171.9
**L-2–Nal[3]**		
α	4.78 td (3.6, 8.4 Hz)	54.0
β, β′	3.20 dd (3.6, 13.8 Hz), 3.09 dd (10.2, 13.8 Hz)	37.2
1, 4	7.19* m	129.2*
2	-	137.7*
3,5	7.20 m	128.1
6	7.12 m	126.3
7, 8	7.19* m	129.2*
9,10	-	131.0*
NH	8.38 d (8.4 Hz)	-
C=O	-	171.1
**2-Furoyl[4]**		
C	-	147.3
α	7.05 d (3.6 Hz)	114.6
β	6.54 dd (1.8, 3.6 Hz)	111.9
γ	7.74 br. s	145.3
C=O	-	157.7

5

Residue	δ_H	δ_C
**L-Ala[1]**		
α	4.22 p (7.2 Hz)	48.3
CH_3	1.23 d (7.2 Hz)	18.0
NH	7.60 br. s	-
C=O	-	174.3
NH_2	7.0 br. s, 8.1 d (7.8 Hz)	-
**L-2-Nal[2]**		
α	4.65 td (4.2, 8.4 Hz)	63
β, β′	3.13 dd (4.2, 13.8 Hz), 3.07 dd (8.4, 13.8 Hz)	51
1	7.75* m	127*
2	-	133
3	7.75* m	127*
4	7.75* m	128*
5, 8	7.71* m	126
6, 7	7.71* m	128*
9	-	134
10	-	133
NH	8.29 d (7.8 Hz)	-
C=O	-	170.3
**L-2–Nal[3]**		
α	4.73 td (4.2, 9.0 Hz)	54.1
β, β′	3.01 dd (9.0, 13.8 Hz), 3.25 dd (4.2, 13 Hz)	37.3
1	7.42 m	128*
2	-	134
3	7.40 m	125.6
4	7.70* m	127.5
5, 8	7.39 m	125.1
6, 7	7.39 m	127.2
9	-	133.0
10	-	132
NH	8.30 d (8.4 Hz)	-
C=O	-	170.0
**2-Furoyl[4]**		
C	-	147.3
α	7.05 d (3.0 Hz)	114.6
β	6.53 dd (1.8, 3.0 Hz)	112.1
γ	7.76 br. s	145.3
C=O	-	157.7

Table S2: ^{1}H-NMR (600 MHz) and ^{13}C-NMR (150 MHz) data of conjugate 6-10 in DMSO-d_6

6			7			8			9			10		
**Residue**	**δ_H**	**δ_C**	**Residue**	**δ_H**	**δ_C**	**Residue**	**δ_H**	**δ_C**	**Residue**	**δ_H**	**δ_C**	**Residue**	**^{1}H (δ, ppm)**	**δ_C**
**L-Ala^{1}**			**L-Phe^{1}**			**L-2–Nal^{1}**			**L-Ala^{1}**			**L-Ala^{1}**		
α	4.19 p (7.2 Hz)	48.2	α	4.44 td (4.8, 8.4 Hz)	54.2	α	4.45 td (4.8, 9 Hz)	53.9	α	4.18 p (7.2 Hz)	48.2	α	4.23 p (7.2 Hz)	48.5
CH_3	1.23 d (7.2 Hz)	18.4	β, β'	2.85 dd (9, 13.8 Hz), 3.05 dd (4.8, 13.8 Hz)	37.5	β, β'	3.15 dd (4.8, 13.2 Hz), 2.98 dd (9, 13.2 Hz)	37.8	CH_3	1.20 d (7.2 Hz)	18.4	CH_3	1.17 d (7.2 Hz)	18.6
NH	8.22 d (7.2 Hz)	-	1	-	135.4	1	7.42 m	128.3	NH	8.04 d (7.2 Hz)	-	NH	8.37 d (7.2 Hz)	-
NH_2	7.78 br. s	-	2, 6	7.60 m	127.6	2	-	137.9	NH_2	7.80 d (7.2 Hz)	-	NH_2	7.80 br. s	-
C=O	-	174.2	3, 5	7.30 m	126.5	3	7.31 m	128.1	C=O	-	174.3	C=O	-	174.7
**L-2–Nal^{2}**			4	7.10 m	126.0	4	7.77 m	125.6	**L-Tyr^{2}**			**L-Phg^{2}**		
α	4.65* m	54.0	NH	8.01 d (8.4 Hz)	-	5, 8	7.41 m	125*	α	4.47 td (4.2, 8.4 Hz)	54.4	α	5.54 d (7.8 Hz)	56.6
β, β'	2.95 m, 2.87 m	36.9	C=O	-	173.0	6, 7	7.78 m	126	β, β'	2.70 dd (8.4, 13.8 Hz), 2.92 (4.2, 13.8 Hz)	36.7	1	-	135.9
1	7.38 m	125.5	NH_2	7.80 d (7.8 Hz)	-	9	-	133.1	1	--	130.3	2, 6	7.40* m	127.7
2	-	137.9	**L-2–Nal^{2}**			10	-	131.9	2, 6	7.00* m	127.6	3, 5	7.70 m	126.1
3	7.70 m	127.5	α	4.55 td (4.8, 8.4 Hz)	54.1	NH	7.99 d (8.4 Hz)	-	3, 5	7.00* m	127.9	4	7.40* m	126.1
4	7.40 m	125.6	β, β'	2.96 dd (9, 13.8 Hz), 3.11 dd (4.8, 13.8 Hz)	37.7	NH_2	7.82 br. s	-	4	-	155.9	NH	8.69 d (7.8 Hz)	-
5, 8	7.19 m	128.1	1	7.65* m	128.1*	C=O	-	174.5	NH	8.24 d (7.8 Hz)	-	C=O	-	169.5
6, 7	7.25 m	129.2	2	-	137.9	**L-Phe^{2}**			OH	9.13 br. s	-	**L-2–Nal^{3}**		
9	-	133.0	3	7.65* m	128.1*	α	4.53 td (5.5, 8.4 Hz)	54.2	C=O	-	171.1	α	4.92 td (4.2, 10.2 Hz)	54.3
10	-	131.0	4	7.40 m	125.6	β, β'	2.73 dd (9, 13.8 Hz), 2.90 dd (4.8, 13.8 Hz)	37.8	**L-2–Nal^{3}**			β, β'	3.14 m, 3.18 m	37.6
NH	8.09 d (7.2 Hz)	-	5, 8	7.35 m	127.5	1	-	135.4	α	4.75 td (4.2, 9.6 Hz)	54.1	1	7.26 m	128.4
C=O		171.1	6, 7	7.72 m	129.3	2, 6	7.07 m	124	β, β'	3.06 dd (10.2, 13.8 Hz), 3.15 dd (4.2, 13.8 Hz)	37.3	2	-	138.30
**L-Phe^{3}**			9	-	133.1	3, 5	7.08 m	123	1	7.38 m	125.5	3	7.05 m	127.8
α	4.65* m	54.1	10	-	131.9	4	7.09 m	125*	2	-	135.7	4	7.15 m	127.7
β, β'	3.22 m, 2.97 m	37.6	NH	8.06 d (7.8 Hz)	-	NH	8.03 d (7.8 Hz)	-	3	7.40 m	125.6	5, 8	7.30 m	128.1
1	--	135.4	C=O	-	172.3	C=O	-	171.5	4	7.70 m	127.5	6, 7	7.40* m	128.9
2, 6	7.15 m	126.5	**L-Ala^{3}**			**L-Ala^{3}**			5, 8	7.19* m	128.1	9	-	133.4
3, 5	7.60 m	127.6	α	4.28 p (7.2 Hz)	48.8	α	4.31 p (7.2 Hz)	48.3	6, 7	7.25 m	129.2	10	-	132.3
4	7.22 m	127.4	CH_3	1.19 d (7.2 Hz)	17.8	CH_3	1.15 d (7.2 Hz)	17.1	9	-	133.0	NH	8.44 d (8.4 Hz)	-
NH	8.27 d (8.4 Hz)	-	NH	8.21 d (7.2 Hz)	-	NH	8.16 d (7.2 Hz)	-	10	-	131.0	C=O		171.5
C=O	-	170.4	C=O	-	170.7	C=O	-	170.1	NH	8.35 d (8.4 Hz)	-	**2-Furoyl^{4}**		
**2-Furoyl^{4}**			**2-Furoyl^{4}**			**2-Furoyl^{4}**			C=O	-	170.8	C	-	147.5
C	-	147.3	C	-	147.3	C	-	147.1	**2-Furoyl^{4}**			α	6.94 br. s	114.5
α	7.02 d (3 Hz)	114.1	α	7.07 br. s	114.3	α	7.12 d (3.6 Hz)	114.1	C	-	147.3	β	6.55 dd (1.8, 3.6 Hz)	112.5
β	6.57 dd (1.2, 3 Hz)	112	β	6.6 dd (1.8, 3.6 Hz)	112.9	β	6.61 dd (1.8, 3.6 Hz)	112	α	7.19* br. s	114.1	γ	7.70 br. s	145.8
γ	7.77 br. s	145.3	γ	7.75 br. s	145.4	γ	7.78 br. s	145.3	β	6.56 dd (1.8, 3.6 Hz)	112.1	C=O	-	158.4
C=O	-	157.6	C=O	-	157.8	C=O	-	158.4	γ	7.74 br. s	145.3			
									C=O	-	157.7			

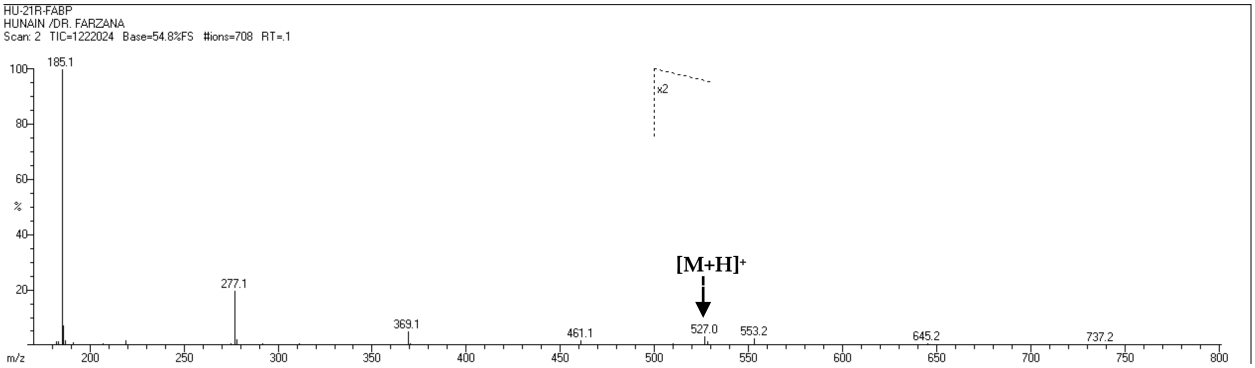

Figure S4: FABMS (Low Resolution Positive Mode) of Conjugate 1

HEJ-MASS LAB -ICCBS				JEOL HX 110 MASS SPECTROMETER (FAB-HR)			
STUDENT NAME		HUNAIN ALI		SAMPLE CODE		DATE	14-11-17
SUPERVISOR NAME		Prof.Dr. Farzana Shaheen		HU-21R		FAB (+VE / -VE)	FAB +VE
Mass	Theoretical Mass	Delta [ppm]	Delta [mmu]	RDB	Composition		
527.2305	527.2308	-0.5	-0.3	17	C32 H33 O6 N1		
	527.2294	2	1.1	17.5	C30 H31 O5 N4		
	527.2326	-4.1	-2.1	4	C20 H37 O13 N3		
	527.2281	4.5	2.4	12.5	C29 H35 O9		
	527.2335	-5.6	-3	21.5	C35 H31 O3 N2		
	527.234	-6.6	-3.5	3.5	C22 H39 O14		
	527.2268	7.1	3.7	13	C27 H33 O8 N3		
	527.2353	-9.1	-4.8	8.5	C23 H35 O10 N4		

Figure S5: FABMS (High Resolution Positive Mode) of Conjugate 1

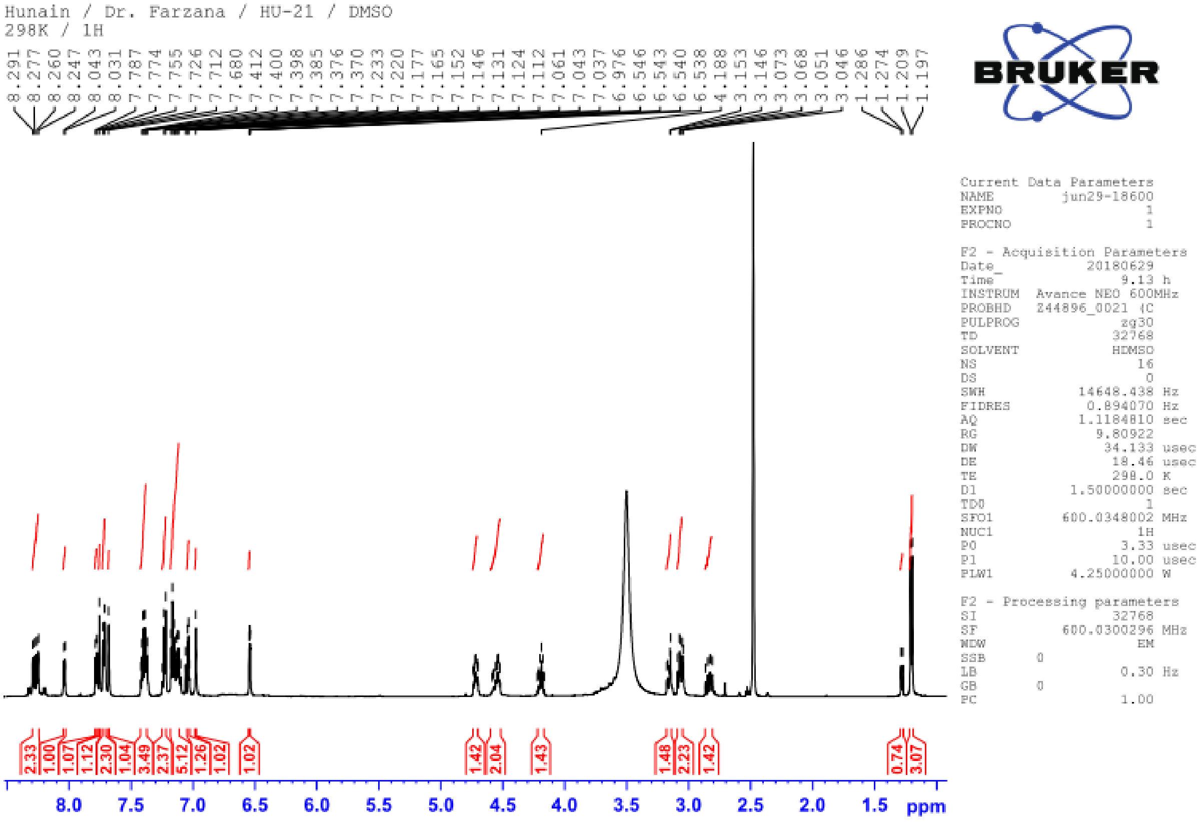

Figure S6: ^{1}H-NMR of Conjugate 1

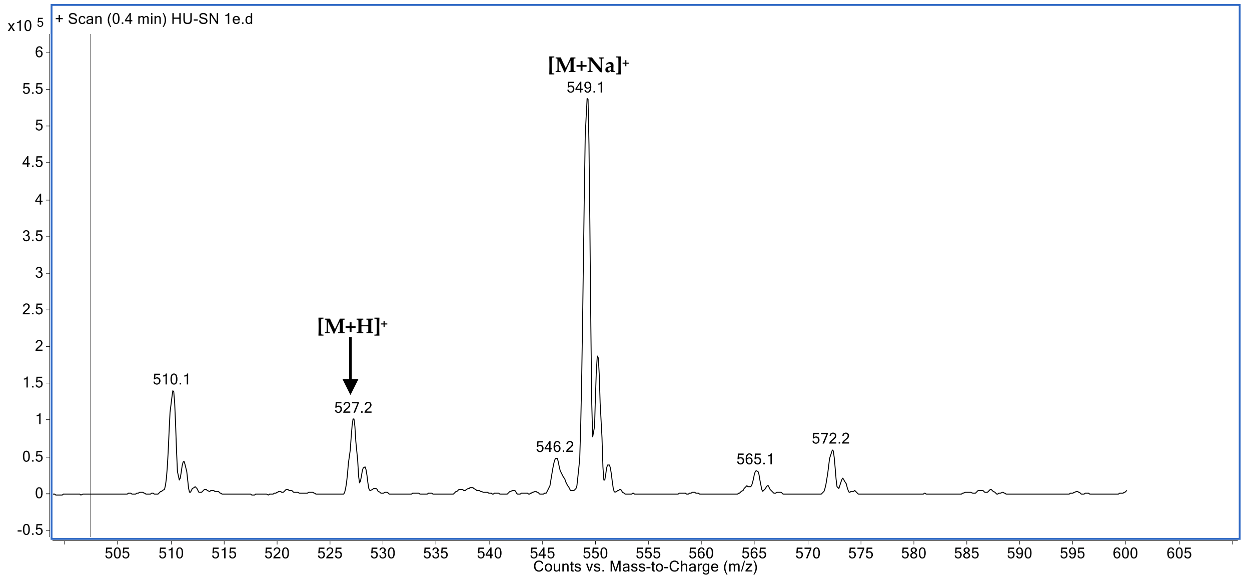

Figure S7: ESIMS (Low Resolution Positive Mode) of Conjugate 2

HEJ-MASS LAB -ICCBS				JEOL HX 110 MASS SPECTROMETER (FAB-HR)			
STUDENT NAME		HUNAIN ALI		SAMPLE CODE		DATE	21-7-17
SUPERVISOR NAME		Prof.Dr. Farzana Shaheen		HU-SN-1e		FAB (+VE / -VE)	FAB +VE
Mass	Theoretical Mass	Delta [ppm]	Delta [mmu]	RDB	Composition		
527.2311	527.2308	0.6	0.3	17	C32 H33 O6 N1		
	527.2294	3.1	1.7	17.5	C30 H31 O5 N4		
	527.2335	-4.5	-2.4	21.5	C35 H31 O3 N2		
	527.2281	5.7	3	12.5	C29 H35 O9		
	527.2353	-8	-4.2	8.5	C23 H35 O10 N4		
	527.2268	8.2	4.3	13	C27 H33 O8 N3		

Figure S8: FABMS (High Resolution Positive Mode) of Conjugate 2

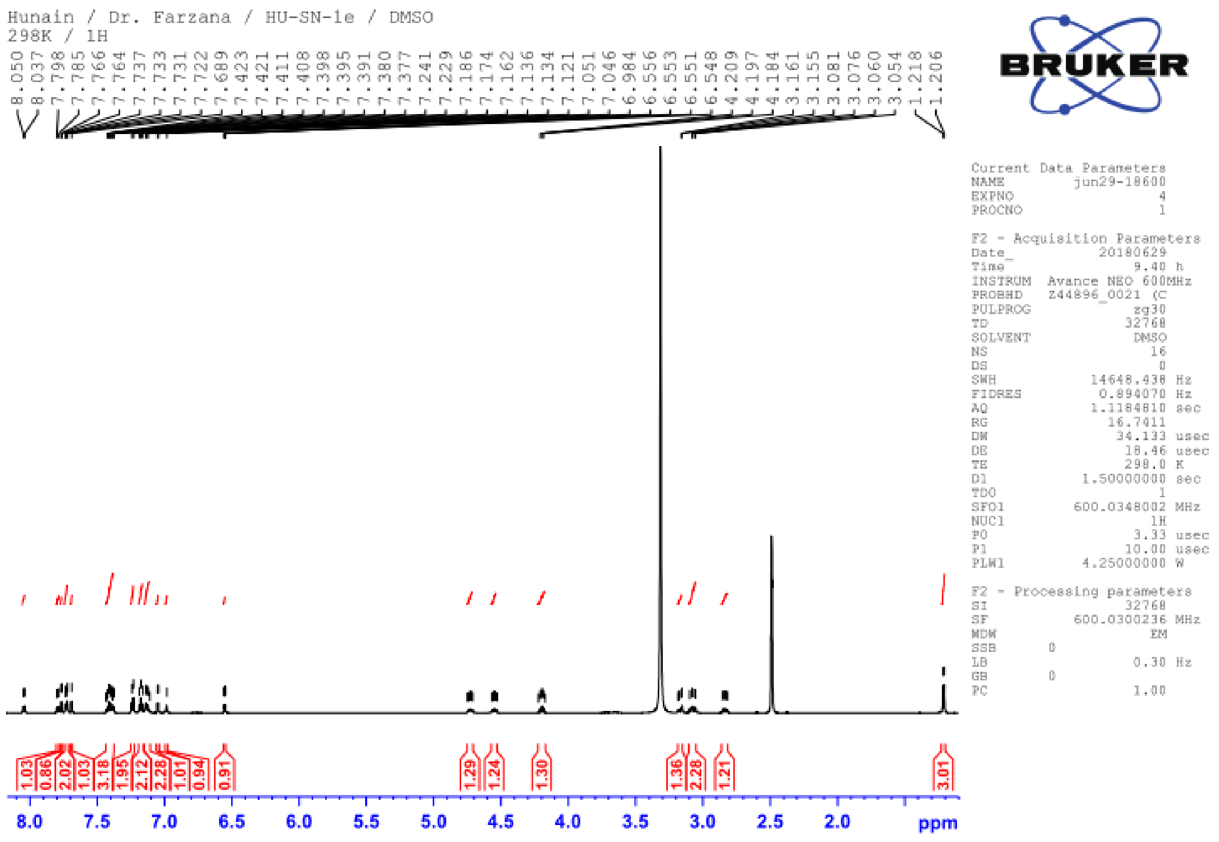

Figure S9: ^{1}H-NMR of Conjugate 2

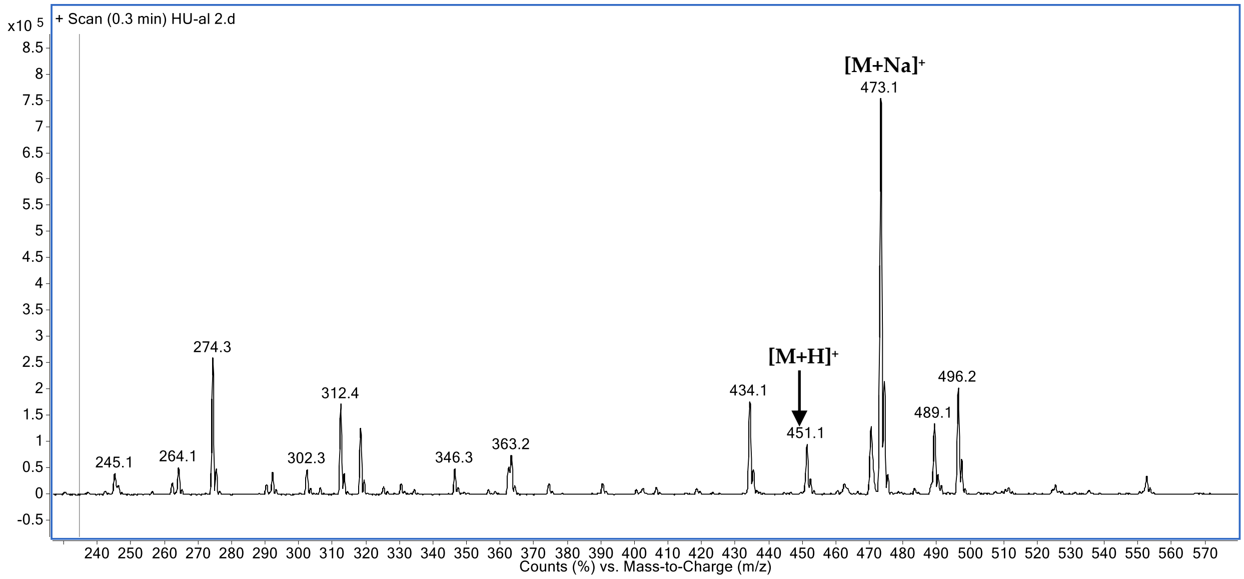

Figure S10: ESIMS (Low Resolution Positive Mode) of Conjugate 3

HEJ-MASS LAB -ICCBS				JEOL HX 110 MASS SPECTROMETER (FAB-HR)			
STUDENT NAME	HUNAIN ALI			SAMPLE CODE		DATE	21-7-17
SUPERVISOR NAME	Prof. Dr. Farzana Shaheen			HU-SAL-2		FAB (+VE / -VE)	FAB +VE
Mass	Theoretical Mass	Delta [ppm]	Delta [mmu]	RDB	Composition		
451.19800	451.1981	-0.3	-0.1	13.5	C24 H27 O5 N4		
	451.1968	2.6	1.2	8.5	C23 H31 O9		
	451.1995	-3.3	-1.5	13	C26 H29 O6 N1		
	451.1955	5.6	2.5	9	C21 H29 O8 N3		
	451.2008	-6.3	-2.8	18	C27 H25 O2 N5		

Figure S11: FABMS (High Resolution Positive Mode) of Conjugate 3

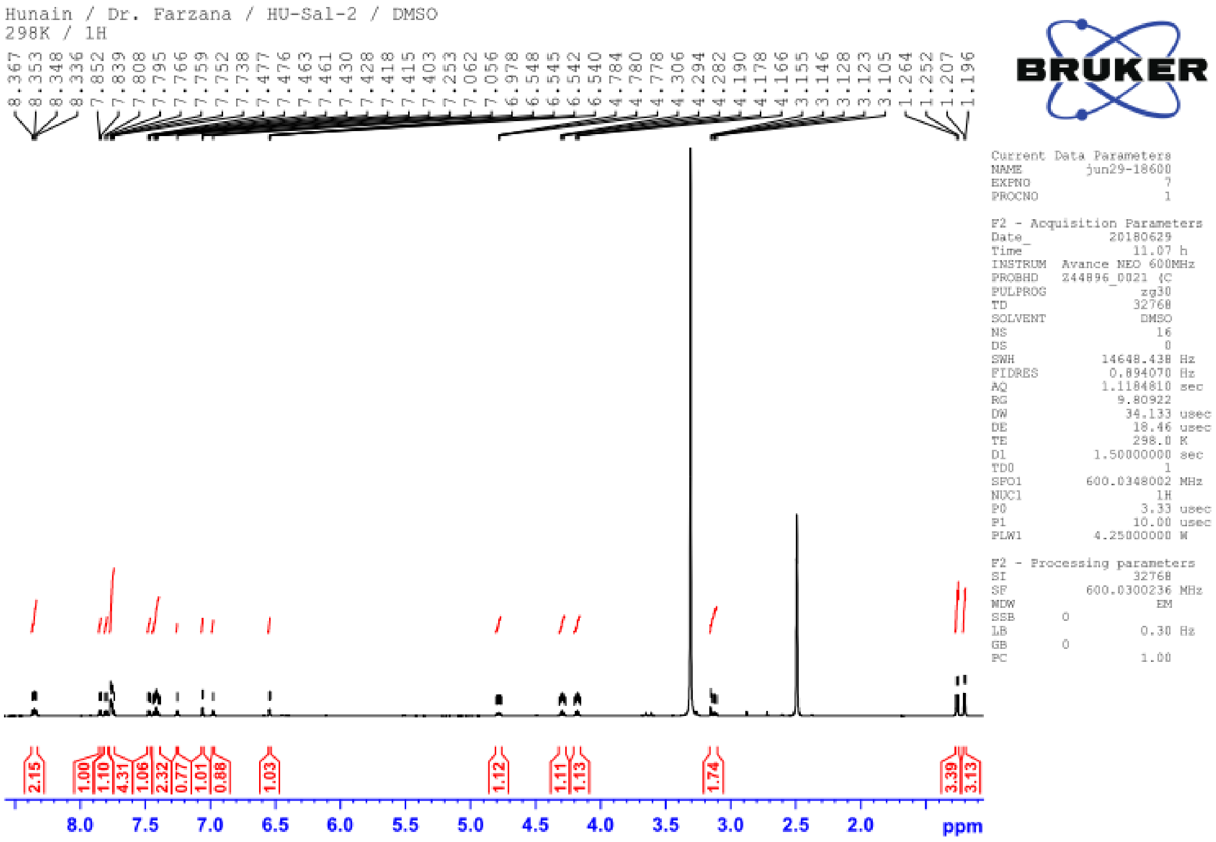

Figure S12: ¹H-NMR of Conjugate 3

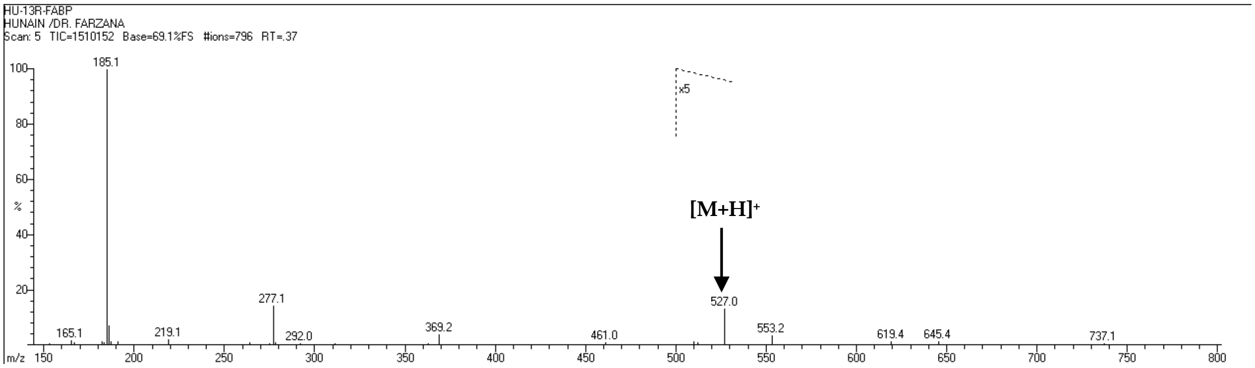

Figure S13: FABMS (Low Resolution Positive Mode) of Conjugate 4

HEJ-MASS LAB -ICCBS				JEOL HX 110 MASS SPECTROMETER (FAB-HR)			
STUDENT NAME		HUNAIN ALI		SAMPLE CODE		DATE	14-11-17
SUPERVISOR NAME		Prof.Dr. Farzana Shaheen		HU-13R		FAB (+VE / -VE)	FAB +VE
Mass	Theoretical Mass	Delta [ppm]	Delta [mmu]	RDB	Composition		
527.2299	527.2294	0.9	0.5	17.5	C30 H31 O5 N4		
	527.2308	-1.7	-0.9	17	C32 H33 O6 N1		
	527.2281	3.4	1.8	12.5	C29 H35 O9		
	527.2326	-5.2	-2.7	4	C20 H37 O13 N3		
	527.2268	5.9	3.1	13	C27 H33 O8 N3		
	527.2335	-6.8	-3.6	21.5	C35 H31 O3 N2		
	527.234	-7.7	-4.1	3.5	C22 H39 O14		
	527.2249	9.5	5	26	C39 H29 O1 N1		

Figure S14: FABMS (High Resolution Positive Mode) of Conjugate 4

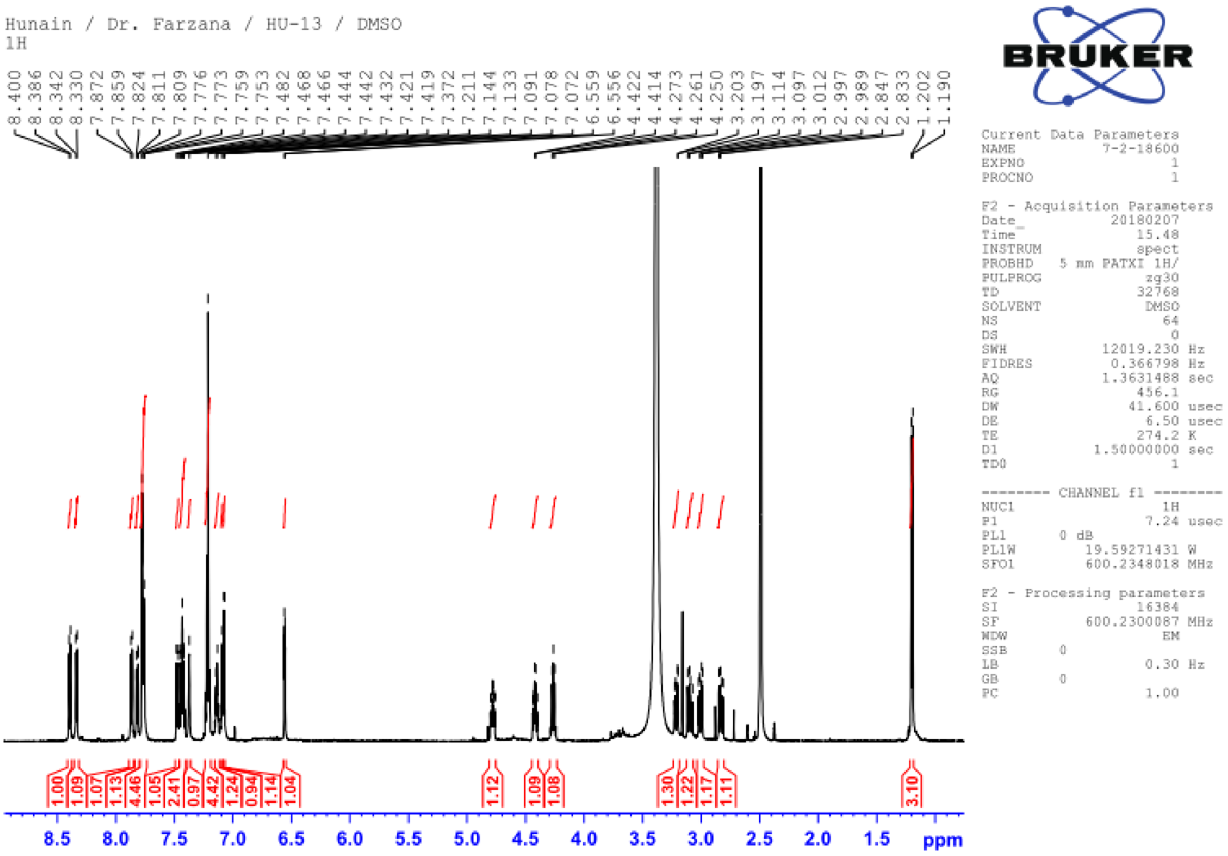

Figure S15: ¹H-NMR of Conjugate 4

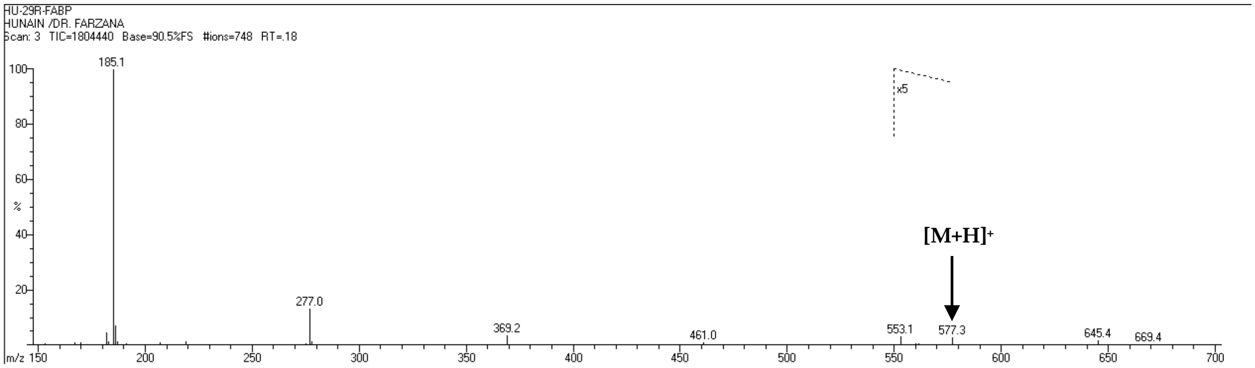

Figure S16: FABMS (Low Resolution Positive Mode) of Conjugate 5

HEJ-MASS LAB -ICCBS				JEOL HX 110 MASS SPECTROMETER (FAB-HR)			
STUDENT NAME		HUNAIN ALI		SAMPLE CODE		DATE	15-11-17
SUPERVISOR NAME		Prof.Dr. Farzana Shaheen		HU-29R		FAB (+VE / -VE)	FAB +VE
Mass	Theoretical Mass	Delta [ppm]	Delta [mmu]	RDB	Composition		
577.2472	577.2464	1.3	0.8	20	C36 H35 O6 N1		
	577.2491	-3.3	-1.9	24.5	C39 H33 O3 N2		
	577.2451	3.6	2.1	20.5	C34 H33 O5 N4		
	577.2438	6	3.4	15.5	C33 H37 O9		
	577.251	-6.5	-3.8	11.5	C27 H37 O10 N4		
	577.2518	-8	-4.6	29	C42 H31 N3		
	577.2424	8.3	4.8	16	C31 H35 O8 N3		
	577.2531	-10.3	-5.9	28.5	C44 H33 O1		
	577.2406	11.5	6.6	29	C43 H31 O1 N1		
	577.255	-13.5	-7.8	15.5	C32 H37 O8 N2		

Figure S17: FABMS (High Resolution Positive Mode) of Conjugate 5

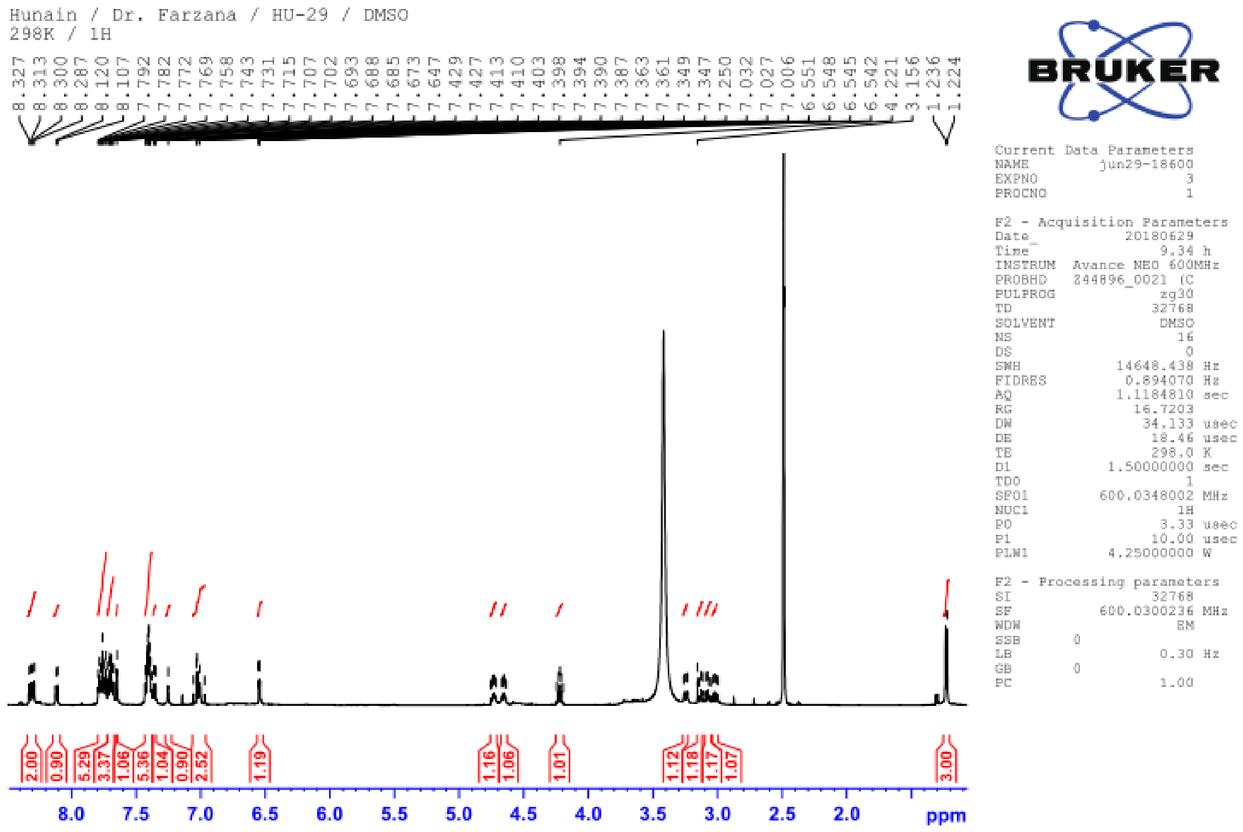

Figure S18: ¹H-NMR of Conjugate 5

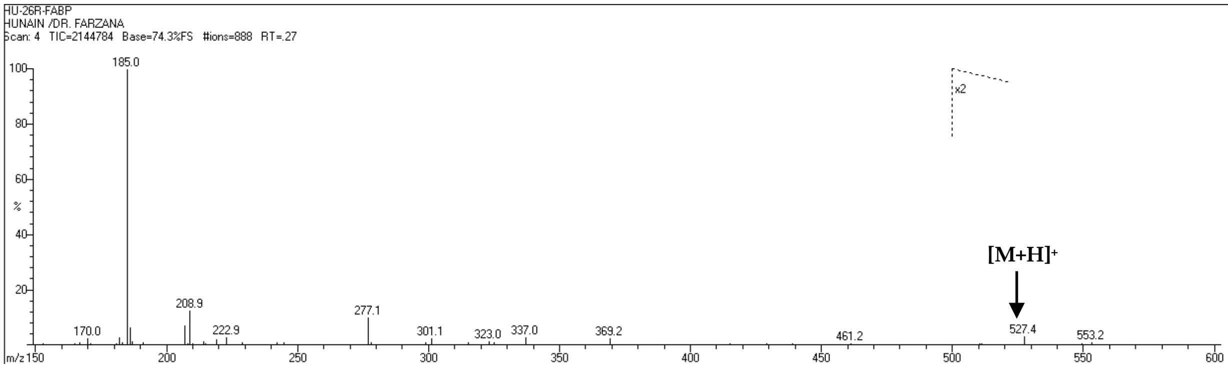

Figure S19: FABMS (Low Resolution Positive Mode) of Conjugate 6

HEJ-MASS LAB -ICCBS				JEOL HX 110 MASS SPECTROMETER (FAB-HR)			
STUDENT NAME		HUNAIN ALI		SAMPLE CODE		DATE	15-11-17
SUPERVISOR NAME		Prof.Dr. Farzana Shaheen		HU-26R		FAB (+VE / -VE)	FAB +VE
Mass	Theoretical Mass	Delta [ppm]	Delta [mmu]	RDB	Composition		
527.2309	527.2308	0.2	0.1	17	C32 H33 O6 N1		
	527.2294	2.8	1.5	17.5	C30 H31 O5 N4		
	527.2335	-4.9	-2.6	21.5	C35 H31 O3 N2		
	527.2281	5.3	2.8	12.5	C29 H35 O9		
	527.2268	7.8	4.1	13	C27 H33 O8 N3		
	527.2353	-8.4	-4.4	8.5	C23 H35 O10 N4		
	527.2361	-10	-5.2	26	C38 H29 N3		
	527.2249	11.4	6	26	C39 H29 O1 N1		
	527.2375	-12.5	-6.6	25.5	C40 H31 O1		
	527.2236	13.9	7.3	26.5	C37 H27 N4		

Figure S20: FABMS (High Resolution Positive Mode) of Conjugate 6

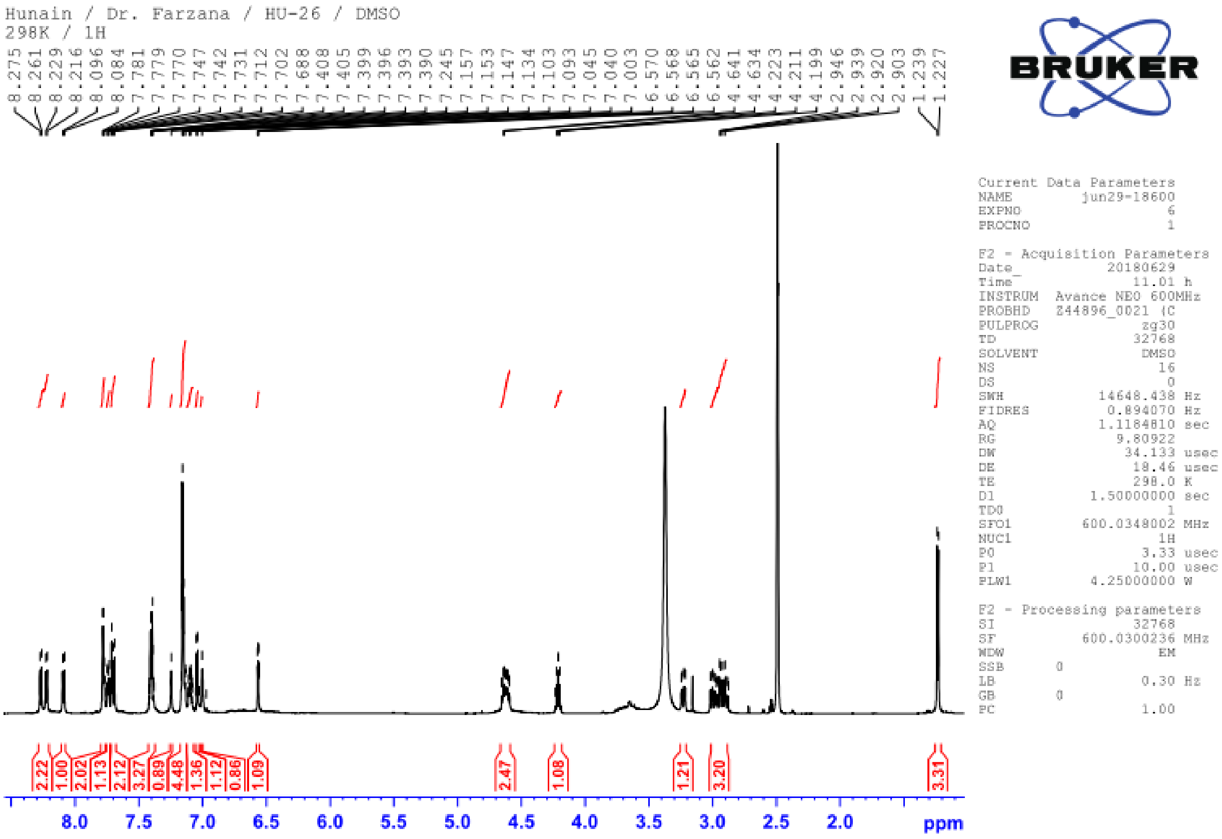

Figure S21: ¹**H-NMR of Conjugate 6**

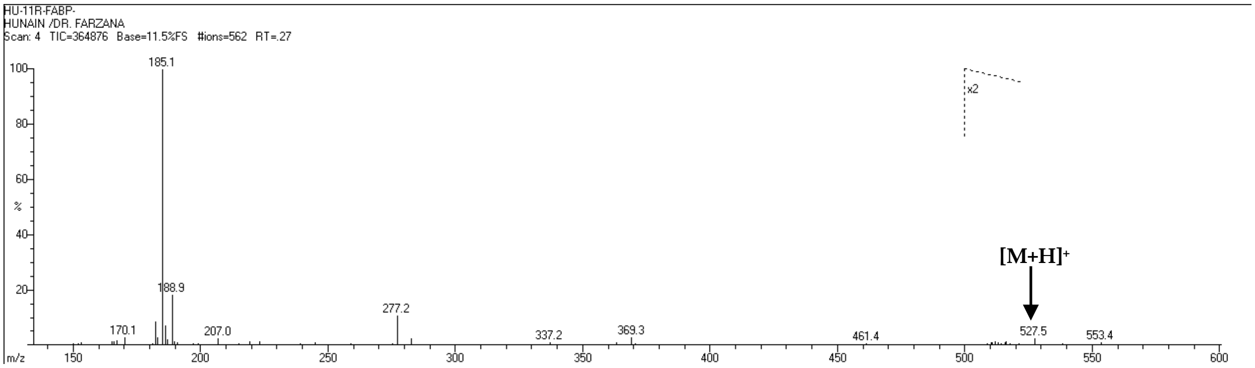

Figure S22: FABMS (Low Resolution Positive Mode) of Conjugate 7

HEJ-MASS LAB -ICCBS				JEOL HX 110 MASS SPECTROMETER (FAB-HR)			
STUDENT NAME		HUNAIN ALI		SAMPLE CODE		DATE	15-11-17
SUPERVISOR NAME		Prof.Dr. Farzana Shaheen		HU-11R		FAB (+VE / -VE)	FAB +VE
**Mass**	**Theoretical Mass**	**Delta [ppm]**	**Delta [mmu]**	**RDB**	**Composition**		
527.2311	527.2308	0.6	0.3	17	C32 H33 O6 N1		
	527.2326	-2.9	-1.5	4	C20 H37 O13 N3		
	527.2294	3.1	1.7	17.5	C30 H31 O5 N4		
	527.2335	-4.5	-2.4	21.5	C35 H31 O3 N2		
	527.234	-5.5	-2.9	3.5	C22 H39 O14		
	527.2281	5.7	3	12.5	C29 H35 O9		
	527.2353	-8	-4.2	8.5	C23 H35 O10 N4		
	527.2268	8.2	4.3	13	C27 H33 O8 N3		

Figure S23: FABMS (High Resolution Positive Mode) of Conjugate 7

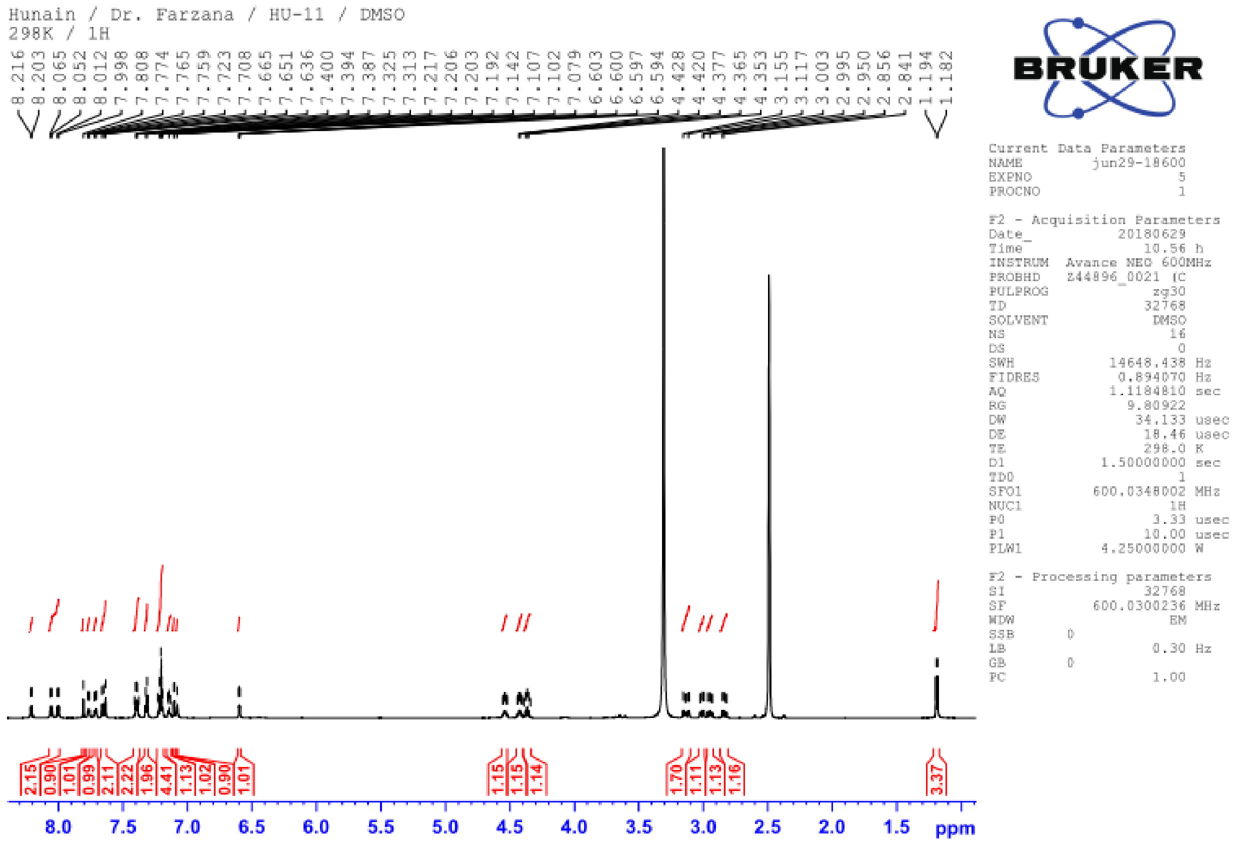

Figure S24: ¹H-NMR of Conjugate 7

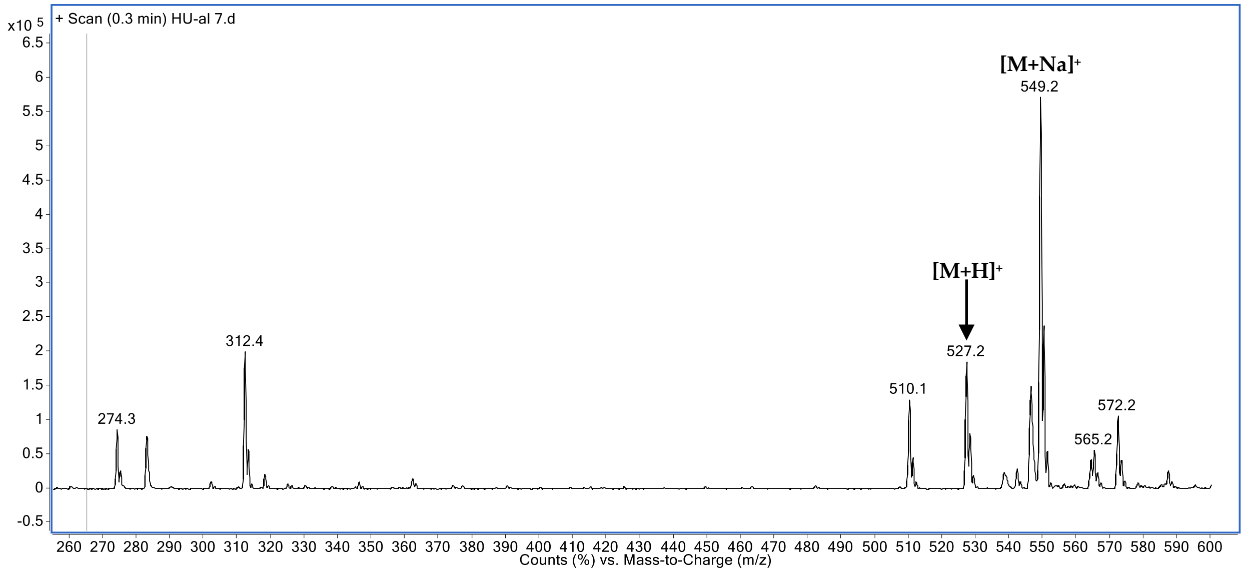

Figure S25: ESIMS (Low Resolution Positive Mode) of Conjugate 8

HEJ-MASS LAB -ICCBS				JEOL HX 110 MASS SPECTROMETER (FAB-HR)			
STUDENT NAME		HUNAIN ALI		SAMPLE CODE		DATE	21-7-17
SUPERVISOR NAME		Prof.Dr. Farzana Shaheen		HU-SAL-7		FAB (+VE / -VE)	FAB +VE
Mass	Theoretical Mass	Delta [ppm]	Delta [mmu]	RDB	Composition		
527.2269	527.2268	0.3	0.1	13	C27 H33 O8 N3		
	527.2281	-2.3	-1.2	12.5	C29 H35 O9		
	527.2249	3.8	2	26	C39 H29 O1 N1		
	527.2294	-4.8	-2.5	17.5	C30 H31 O5 N4		
	527.2236	6.3	3.3	26.5	C37 H27 N4		
	527.2308	-7.4	-3.9	17	C32 H33 O6 N1		
	527.2222	8.8	4.7	21.5	C36 H31 O4		
	527.2209	11.4	6	22	C34 H29 O3 N3		
	527.2335	-12.5	-6.6	21.5	C35 H31 O3 N2		
	527.2353	-16	-8.4	8.5	C23 H35 O10 N4		

Figure S26: FABMS (High Resolution Positive Mode) of Conjugate 8

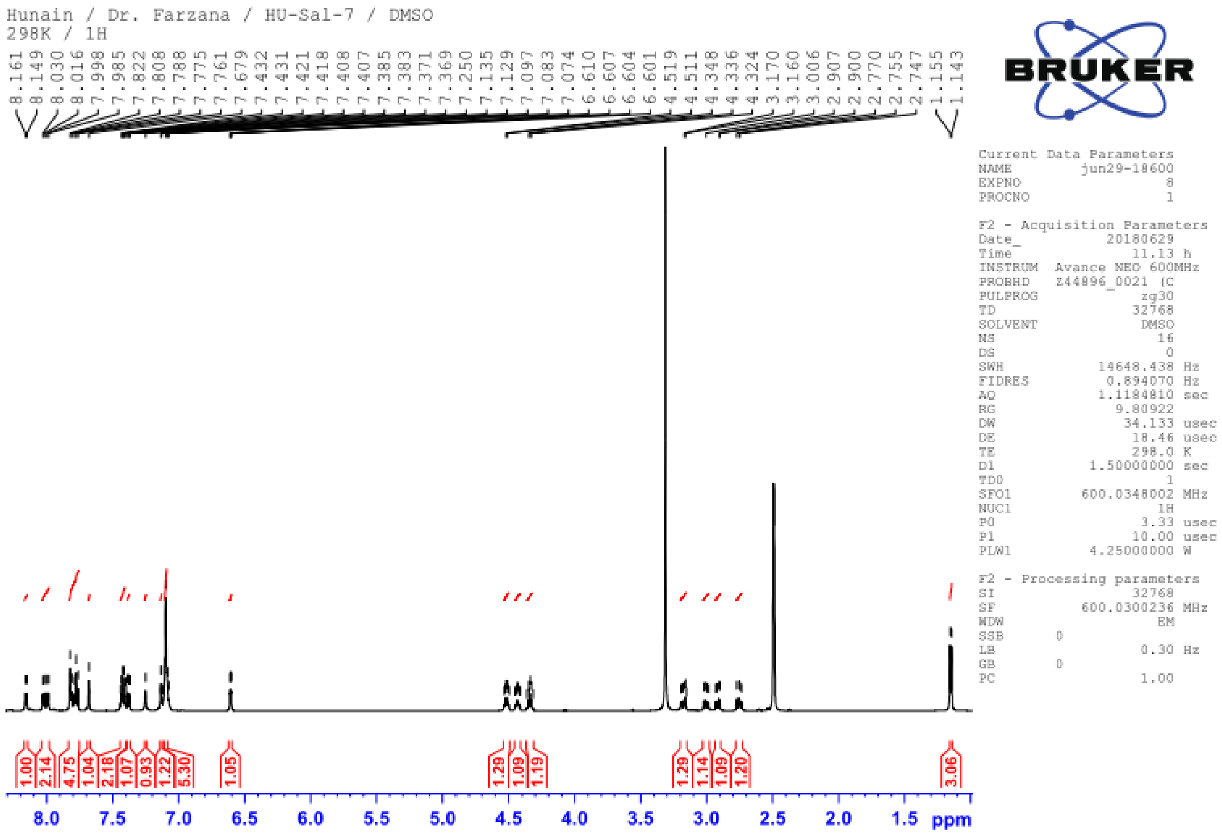

Figure S27: ¹H-NMR of Conjugate 8

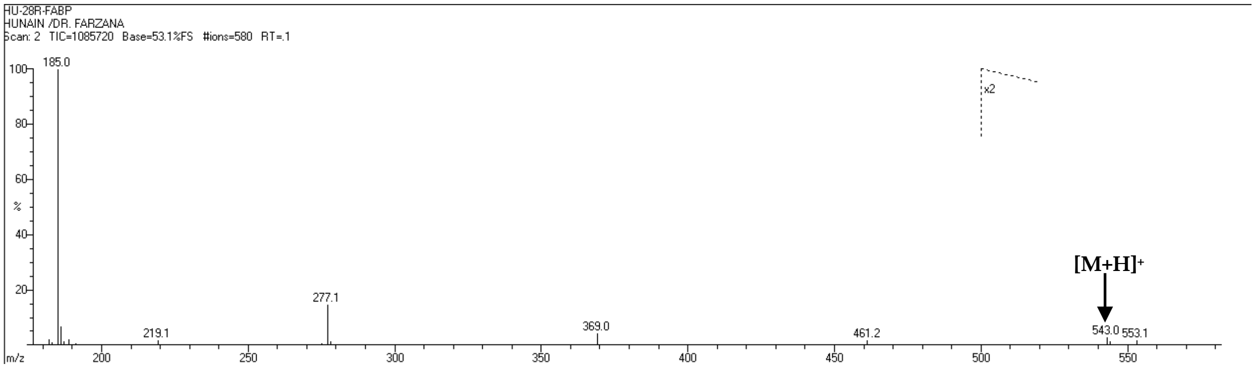

Figure S28: FABMS (Low Resolution Positive Mode) of Conjugate 9

HEJ-MASS LAB -ICCBS				JEOL HX 110 MASS SPECTROMETER (FAB-HR)			
STUDENT NAME	HUNAIN ALI			SAMPLE CODE		DATE	14-11-17
SUPERVISOR NAME	Prof. Dr. Farzana Shaheen			HU-28R		FAB (+VE / -VE)	FAB +VE
Mass	Theoretical	Delta	Delta	RDB	Composition		
	Mass	[ppm]	[mmu]				
543.2256	543.2257	-0.2	-0.1	17	C32 H33 O7 N1		
	543.2244	2.3	1.2	17.5	C30 H31 O6 N4		
	543.2276	-3.6	-2	4	C20 H37 O14 N3		
	543.223	4.7	2.6	12.5	C29 H35 O10		
	543.2284	-5.1	-2.8	21.5	C35 H31 O4 N2		
	543.2289	-6.1	-3.3	3.5	C22 H39 O15		
	543.2217	7.2	3.9	13	C27 H33 O9 N3		
	543.2302	-8.5	-4.6	8.5	C23 H35 O11 N4		

Figure S29: FABMS (High Resolution Positive Mode) of Conjugate 9

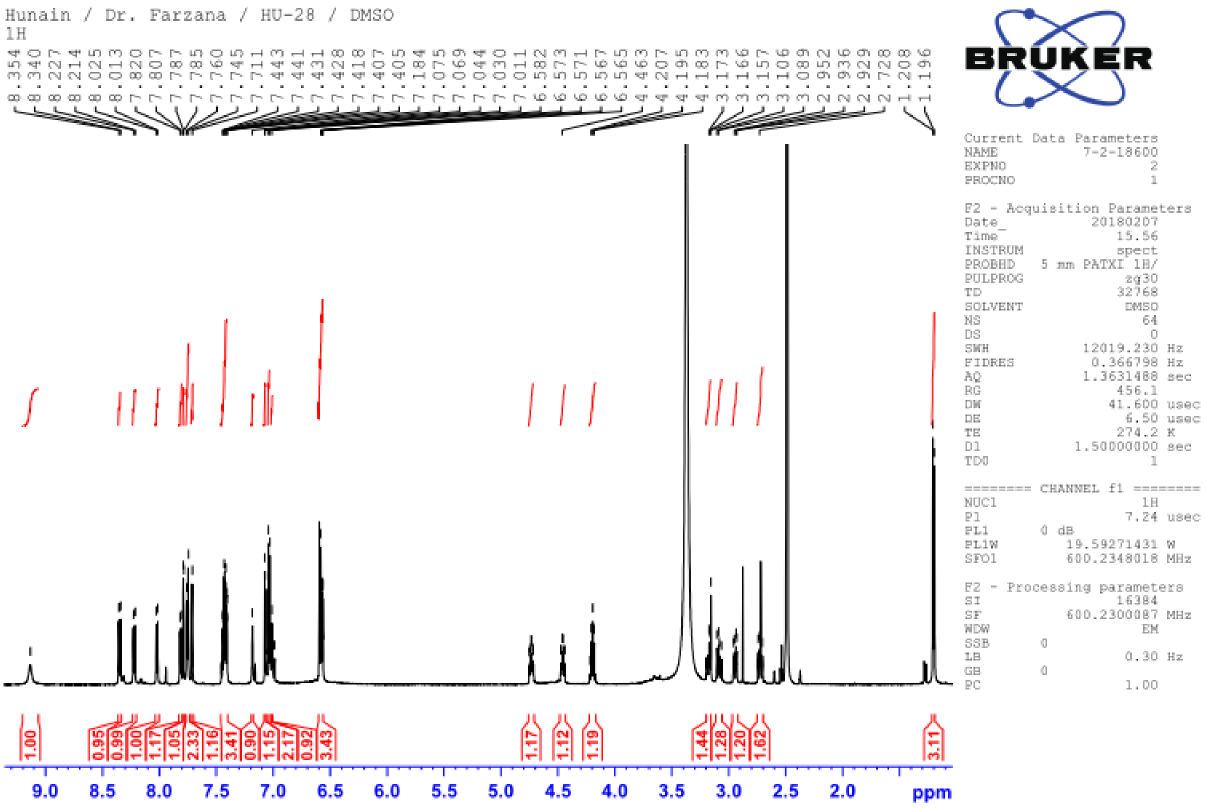

Figure S30: ^{1}H-NMR of Conjugate 9

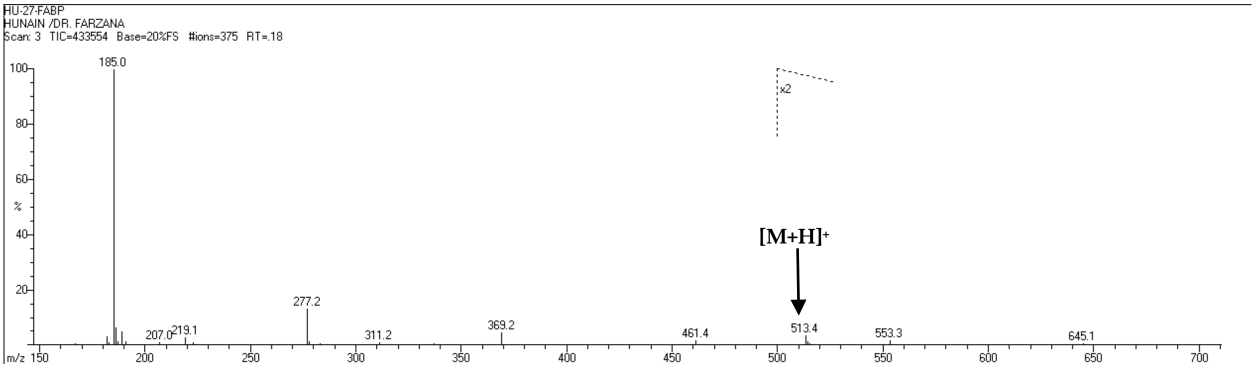

Figure S31: FABMS (Low Resolution Positive Mode) of Conjugate 10

HEJ-MASS LAB -ICCBS				JEOL HX 110 MASS SPECTROMETER (FAB-HR)			
STUDENT NAME		HUNAIN ALI		SAMPLE CODE		DATE	15-11-17
SUPERVISOR NAME		Prof.Dr. Farzana Shaheen		HU-27R		FAB (+VE / -VE)	FAB +VE
Mass	Theoretical Mass	Delta [ppm]	Delta [mmu]	RDB	Composition		
513.2131	513.2125	1.3	0.6	12.5	C28 H33 O9		
	513.2138	-1.4	-0.7	17.5	C29 H29 O5 N4		
	513.2111	3.9	2	13	C26 H31 O8 N3		
	513.2151	-4	-2	17	C31 H31 O6 N1		
	513.2093	7.5	3.8	26	C38 H27 O1 N1		
	513.2178	-9.2	-4.7	21.5	C34 H29 O3 N2		
	513.2079	10.1	5.2	26.5	C36 H25 N4		
	513.2066	12.7	6.5	21.5	C35 H29 O4		
	513.2197	-12.8	-6.6	8.5	C22 H33 O10 N4		
	513.2205	-14.4	-7.4	26	C37 H27 N3		

Figure S32: FABMS (High Resolution Positive Mode) of Conjugate 10

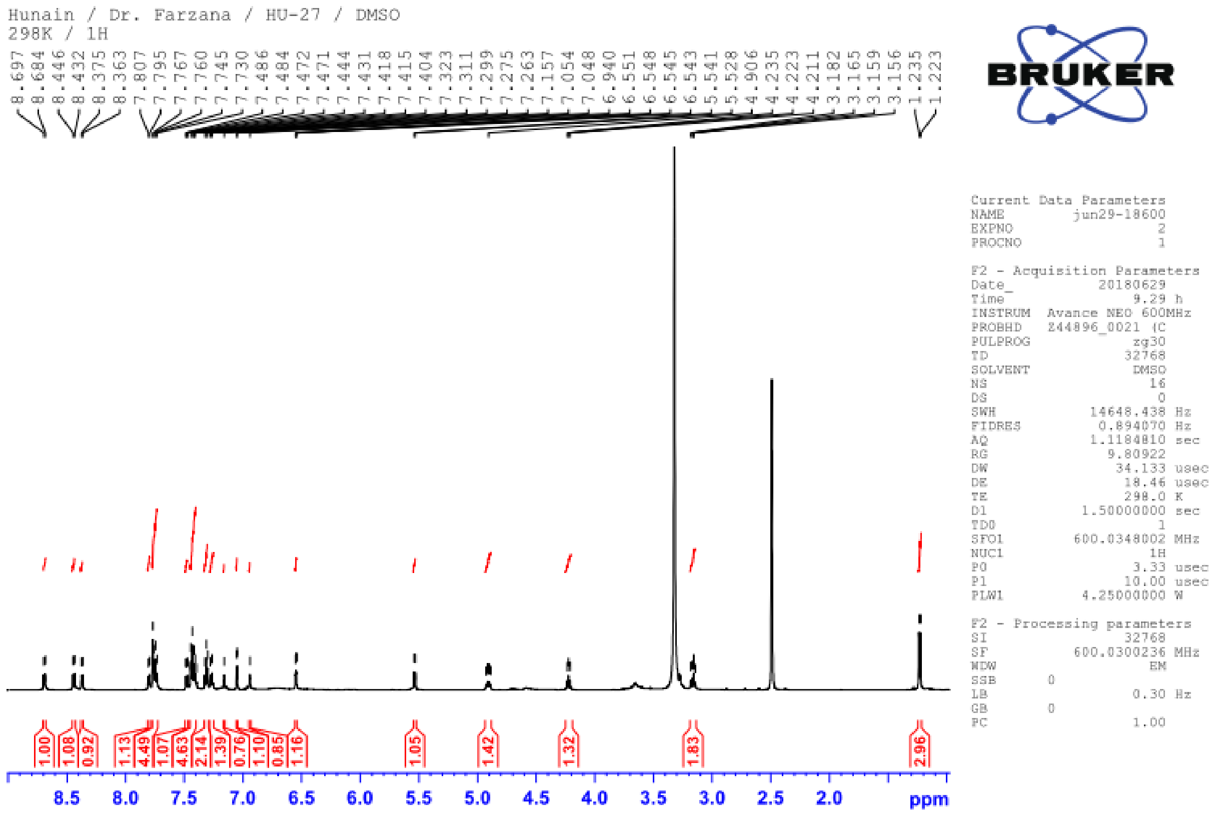

Figure S33: ¹H-NMR of Conjugate 10

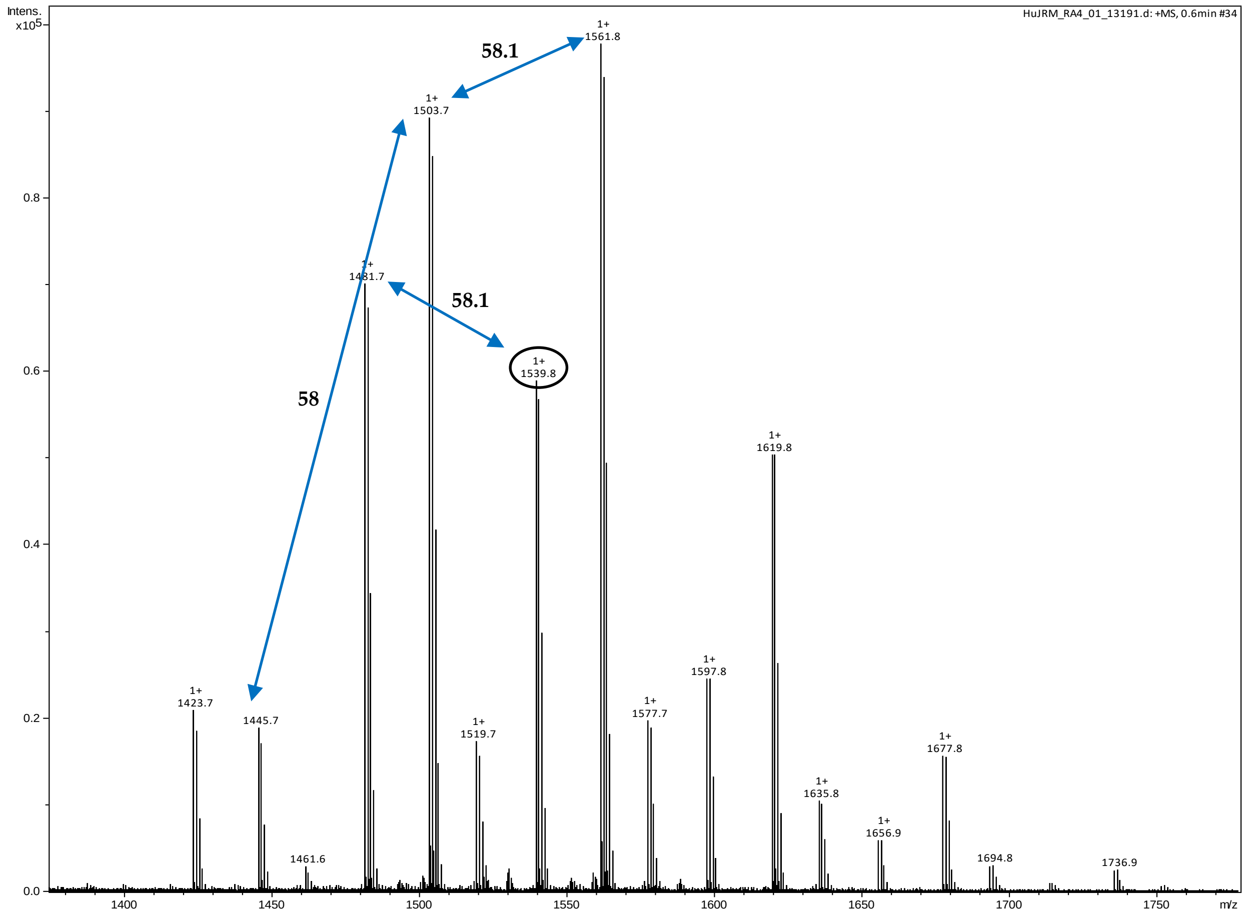

Figure S34: ESI Spectra (Low Resolution) of Conjugate 4a

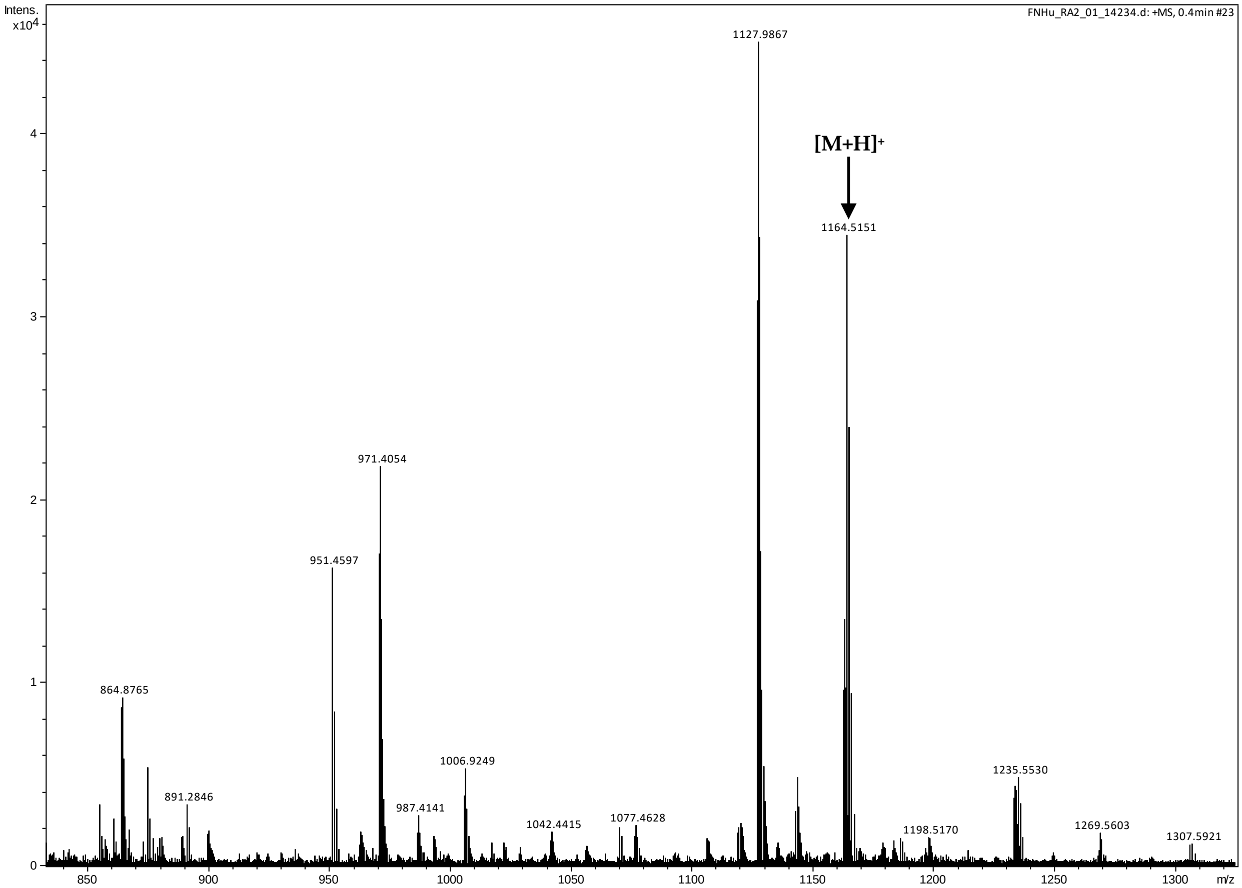

Figure S35: ESI Spectra (Low Resolution) of Conjugate 4b

Table S3: TFA Cocktails for cleavage of peptide conjugates 1-20

S. No.	Protecting groups	TFA cocktail for cleavage	Time given for cleavage
**1**	-	TFA:H_2O::95:5	2 h
**2**	-	TFA:H_2O::95:5	2 h
**3**	-	TFA:H_2O ::95:5	2 h
**4**	-	TFA H_2O ::95:5	2 h
**5**	-	TFA:H_2O::95:5	2 h
**6**	-	TFA:H_2O::95:5	2 h
**7**	-	TFA:H_2O::95:5	2 h
**8**	-	TFA:H_2O::95:5	2 h
**9**	*tBu*	TFA:TIPS:Phenol:H_2O::88:2:5:5	2 h
**10**	-	TFA:H_2O::95:5	2 h
**11**	*tBu*	TFA:DCM:TIPS::95:4:1	2 h
**12**	-	TFA:DCM:TIPS::90: 9:1	2 h
**13**	-	TFA:DCM:TIPS::90:9:1	2 h
**14**	-	TFA:DCM:TIPS:90:9:1	2 h
**15**	-	TFA:DCM:TIPS::95:4:1	2 h
**16**	-	TFA:DCM:TIPS::95:4:1	2 h
**17**	*pbf*	TFA:H_2O:TIPS::90:9:1	2 h
**18**	-	TFA:DCM:TIPS::95:4:1	2 h
**19**	-	TFA:DCM:TIPS::95:4:1	2 h
**20**	-	TFA:H_2O:TIPS::90:9:1	2 h

Table S4: HPLC conditions for peptide conjugates 11-20

S. No	Retention time (min)	HPLC eluted system	Wavelength (nm)
**11**	3.442	ACN: H_2O::85:15	262.9
**12**	3.649	ACN: H_2O::85:15	297.6
**13**	3.365	ACN: H_2O::85:15	253.8
**14**	3.198	ACN: H_2O::85:15	287.7
**15**	3.450	ACN: H_2O::85:15	276.7
**16**	3.407	ACN: H_2O::85:15	267.8
**17**	3.208	ACN: H_2O::85:15	309.4
**18**	3.269	ACN: H_2O::85:15	278.9
**19**	3.642	ACN: H_2O:TFA::85:15:0.08	295.6
**20**	3.415	ACN: H_2O:TFA::85:15:0.08	236.5

Table S5: Sequence and ESIMS (Low Resolution) and FABMS (High Resolution) of peptide conjugates 11-20

S. No.	Sequence	[MH]+, calculated mol. formula	HRMS
11	2-Furoic acid-L-Tyr-L-2 Nal-D-Ala-NH_2	543.22, $C_{30}H_{30}N_4O_6$	543.2278
12	2-Furoic acid-D-Ala-D-Phe-D-Ala-NH_2	401.17, $C_{20}H_{24}N_4O_5$	401.1791
13	2-Furoic acid-D-Ala-D-Phe-Gly-NH_2	387.16, $C_{19}H_{22}N_4O_5$	387.1705
14	2-Furoic acid-D-Ala-D-Phe-L-Lys-NH_2	458.23, $C_{23}H_{31}N_5O_5$	458.2413
15	2-Furoic acid-D-Ala-L-2 Nal-D-Ala-NH_2	451.19, $C_{24}H_{26}N_4O_5$	451.1943
16	2-Furoic acid-D-Ala-L-Ile-D-Ala-NH_2	367.2, $C_{17}H_{28}N_4O_5$	367.2014
17	2-Furoic acid-D-Ala-L-Arg-D-Ala-NH_2	410.21, $C_{17}H_{27}N_7O_5$	410.2143
18	2-Furoic acid- D-Phe-D-Ala-NH_2	329.14, $C_{17}H_{20}N_3O_{4,}$	329.1414
19	2-Furoic acid-L-2-Nal-D-Phe-L-*N*-Me Ala-NH_2	541.2, $C_{31}H_{33}N_4O_5$	541.2120
20	2-Furoic acid-L-2 Nal-D-Phe-L-Ala-NH_2	527.22, $C_{30}H_{30}N_4O_5$	527.2250

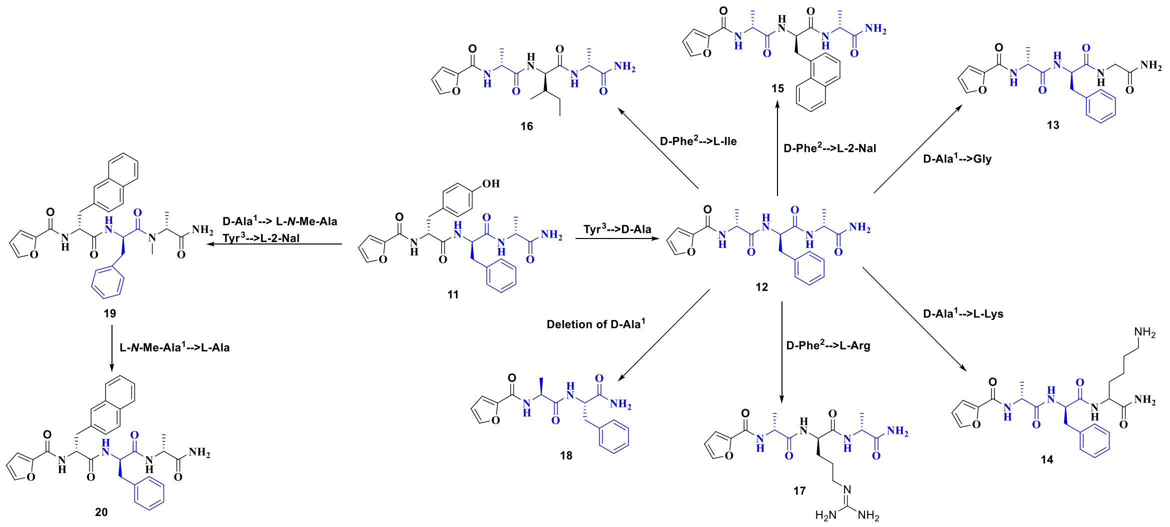

Figure S36: Structural relationship between peptide conjugates 11-20

<table>
<tr><th>Conjugate No.</th><th>ESI-MS Spectrum</th></tr>
<tr><td>11</td><td>
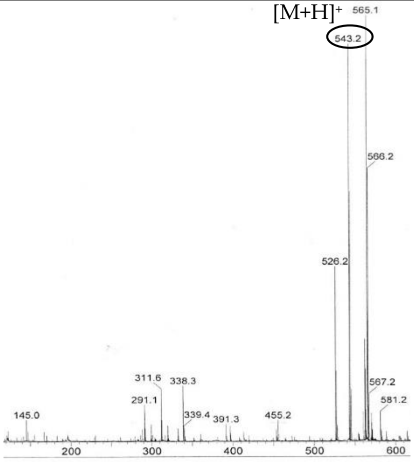

</td></tr>
<tr><td>12</td><td>
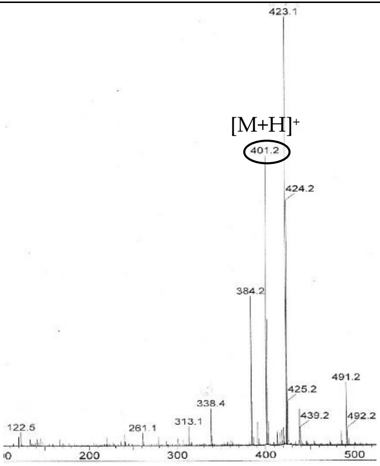

</td></tr>
<tr><td>13</td><td>
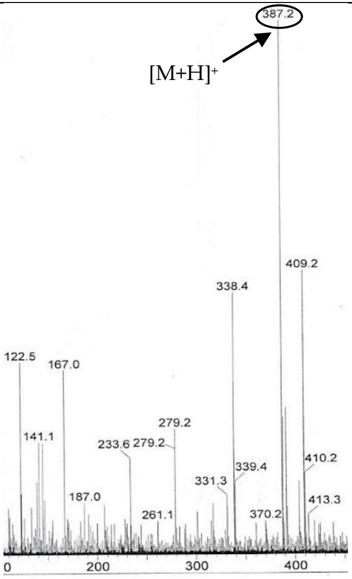

</td></tr>
</table>

**14**	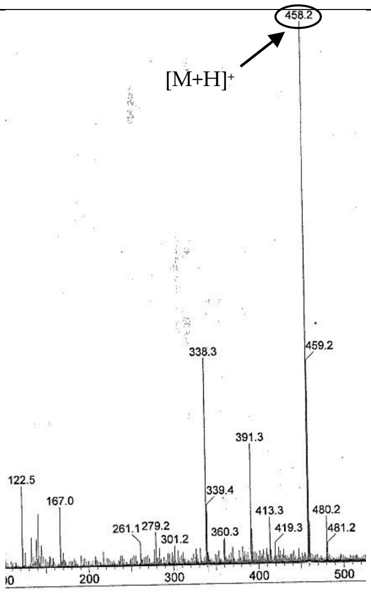458.2<br>[M+H]+<br>338.3<br>459.2<br>391.3<br>122.5<br>167.0<br>339.4<br>413.3<br>480.2<br>261.1<br>279.2<br>301.2<br>360.3<br>419.3<br>481.2<br>200<br>300<br>400<br>500
**15**	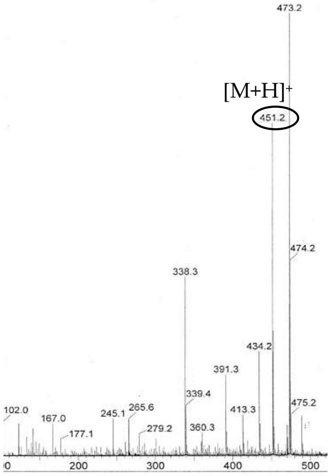473.2<br>[M+H]+<br>451.2<br>474.2<br>338.3<br>434.2<br>391.3<br>339.4<br>102.0<br>167.0<br>245.1<br>265.6<br>413.3<br>475.2<br>177.1<br>279.2<br>360.3<br>200<br>300<br>400<br>500
**16**	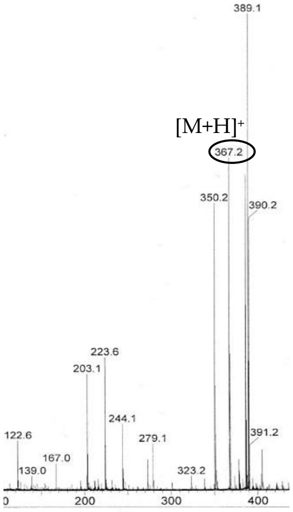389.1<br>[M+H]+<br>367.2<br>350.2<br>390.2<br>223.6<br>203.1<br>244.1<br>122.6<br>279.1<br>391.2<br>167.0<br>139.0<br>323.2<br>200<br>300<br>400

<table>
<tr><td>17</td><td>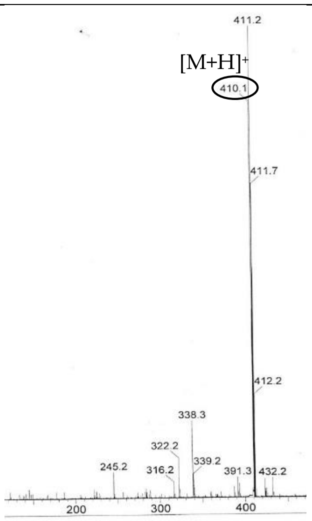
</td></tr>
<tr><td>19</td><td>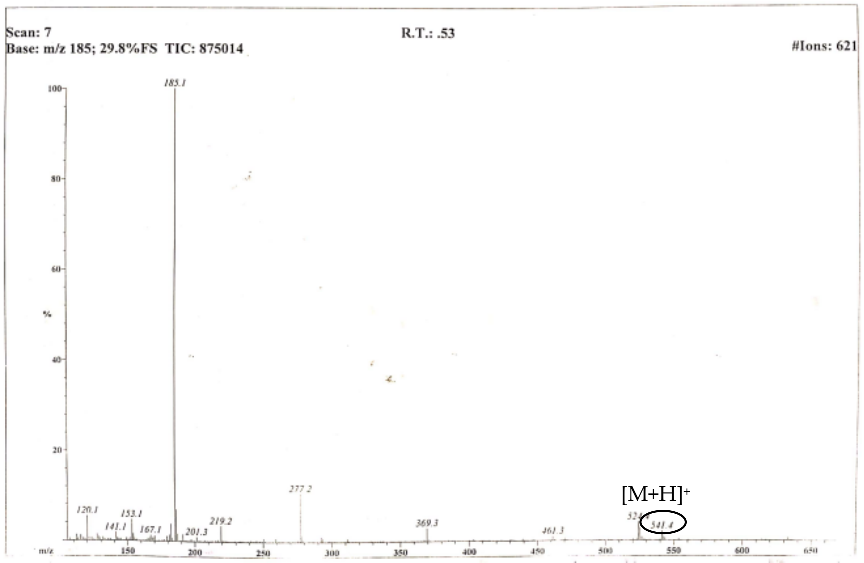
</td></tr>
</table>

**20**	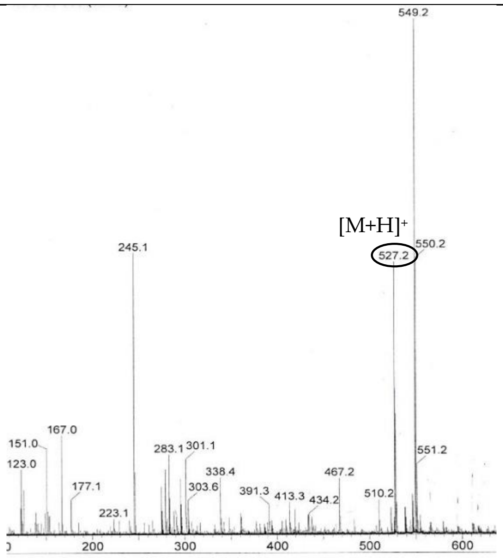

Figure S37: ESI Mass spectrum for peptide conjugates 11-17, 19 (FAB-MS) and 20

© 2020 by the authors. Licensee MDPI, Basel, Switzerland. This article is an open access article distributed under the terms and conditions of the Creative Commons Attribution (CC BY) license (http://creativecommons.org/licenses/by/4.0/).
